# Inter-species Metabolic Interactions in an In-vitro Minimal Human Gut Microbiome of Core Bacteria

**DOI:** 10.1038/s41522-022-00275-2

**Published:** 2022-04-08

**Authors:** Sudarshan A. Shetty, Ben Kuipers, Siavash Atashgahi, Steven Aalvink, Hauke Smidt, Willem M. de Vos

**Affiliations:** 1 Laboratory of Microbiology, Wageningen University & Research, Wageningen, The Netherlands; 2 Department of Microbiology, Radboud University, Nijmegen, The Netherlands; 3 Human Microbiome Research Program, Faculty of Medicine, University of Helsinki, Helsinki, Finland; 4 Present Address: Department of Medical Microbiology and Infection prevention, Virology and Immunology Research Group, University Medical Center Groningen, Groningen, The Netherlands

**Keywords:** Microbial ecology, Microbiome

## Abstract

Knowledge of the functional roles and interspecies interactions are crucial for improving our understanding of the human intestinal microbiome in health and disease. However, the complexity of the human intestinal microbiome and technical challenges in investigating it pose major challenges. In this proof-of-concept study, we rationally designed, assembled and experimentally tested a synthetic Diet-based Minimal Microbiome (Db-MM) consisting of ten core intestinal bacterial species that together are capable of efficiently converting dietary fibres into short chain fatty acids (SCFAs). Despite their genomic potential for metabolic competition, all ten bacteria coexisted during growth on a mixture of dietary fibres, including pectin, inulin, xylan, cellobiose and starch. By integrated analyses of metabolite production, community composition and metatranscriptomics-based gene expression data, we identified interspecies metabolic interactions leading to production of key SCFAs such as butyrate and propionate. While public goods, such as sugars liberated from colonic fibres, are harvested by non-degraders, some species thrive by cross-feeding on energetically challenging substrates, including the butyrogenic conversion of acetate and lactate. Using a reductionist approach in an in-vitro system combined with functional measurements, our study provides key insights into the complex interspecies metabolic interactions between core intestinal bacterial species.

## Introduction

Interactions within microbial communities are highly dynamic and governed by both biotic and abiotic factors. Ecological relationships varying from competition to mutualism and syntrophy are important determinants of community composition and function^1^. An important driver of these relationships is the metabolic potential of the interacting partners, which can result in competition for resources and/or cross-feeding for mutual benefits^2,3^. These interspecies relationships in microbiomes are context dependent, where multiple factors may influence the nature of interactions that occur under a given condition. While pairwise interactions provide insights into emergent behaviours of two strains in presence of each other, the additive effect of additional strains on community compositional and functional dynamics requires more complex communities. These additive effects are often referred to as “higher order interactions” where presence of a third strain may alter pairwise interactions between two strains^4–6^. In competitive networks, higher order interactions may have a stabilizing effect, possibly explaining the coexistence of similar species^7^. Knowledge of metabolic interspecies interactions in presence of several other species with either beneficial or competitive roles is, therefore, crucial for understanding and predicting functioning and stability of microbial communities.

Diet driven interspecies interactions within the intestinal tract, notably the colon, can have a crucial impact on host health. Conversion of dietary fibres that reach the colon undigestedly are converted to short chain fatty acids (SCFAs) via a complex web of metabolic interactions between resident microbial communities^8–10^. These interactions include trophic exchanges between specialized polysaccharide degraders with specialized enzymatic machineries that release simpler substrates (public goods) for other bacteria in the community. Notably, high functional redundancy, i.e. the existence of multiple species with similar functional potential is a hallmark of the intestinal microbiome^9–13^. However, the nature and details of the metabolic strategies employed by species with similar functional potential (likely competitors) that coexist within complex communities with high order interactions remain obscure. Due to practical reasons and the complexity of the human intestinal microbiome, experiments using synthetic defined co-cultures have mainly been used to study cross-feeding and competition for resources^14–17^. Although these co-culture studies have played a key role in improving our understanding of pairwise ecological and metabolic interactions, their intrinsic simplistic nature has overlooked the role of emergent properties and higher order interactions in determining interspecies interactions and community stability. At the highest complexity level, microbial interactions have been investigated by employing a variety of -omics methods to naturally occurring microbiomes, for example, faecal samples^18–20^. However, the high complexity, presence of unknown microbial community members and individual variability of the microbiome have posed major challenges in disentangling interspecies interactions. Reducing the complexity of natural microbiomes by growing a limited number of representative species in defined mixtures (also called minimal microbiomes) has been recently employed for investigating metabolic interactions and ecological processes, such as the assembly of intestinal microbiomes^21–24^.

Here, we leveraged the concept of minimal microbiomes to study interspecies metabolic interactions in presence of common dietary fibres. Notably, when designing the minimal microbiome in our study, we not only consider the species we select, but also the environment we created that consists of multiple substrates, controlled growth conditions and the ensuing interactions. Thus, using this approach allows combining both ecological and physiological features such as competition, cross-feeding and functional redundancy. We name this ecophysiology-guided butyrate- and propionate-producing minimal microbiome as a Diet-based Minimal Microbiome (Db-MM)^25^. We use the term Db-MM to reflect a key design aspect i.e. based on dietary fibres and focuses on the reconstruction of major metabolic routes for conversions of dietary carbohydrates to SCFAs using a ten species minimal microbiome. We combined in-vitro batch cultures, quantitative microbial profiling, metatranscriptomics and metabolite analysis to reconstruct the community-level conversion of dietary fibres to SCFAs. We show that all ten Db-MM strains could coexist for extended periods of time. Using metatranscriptomics, we identified the transcriptional response of each strain and interspecies metabolic interactions that lead to production of butyrate and propionate. By using a defined minimal microbiome, our study unravels and advances our understanding of the metabolic roles and interactions between core bacteria of the intestinal microbiome.

## Results

### Rationale for the design of Db-MM

**Fig. 1:**
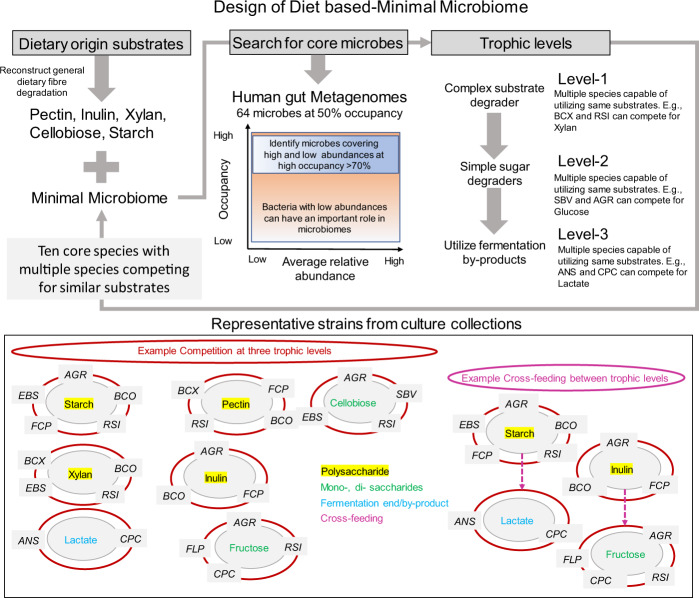
Design of a diet-based minimal microbiome. We aimed to reconstruct the metabolic pathways and species interactions involved in degradation of dietary fibres and production of short-chain fatty acids. Abbreviations: AGR *A. rectalis*, ANS *A. soehngenii*, BCO *B. ovatus*, BCX *B. xylanisolvens*, CPC *C. catus*, EBS *E. siraeum*, FCP *F. prausnitzii*, FLP *F. plautii*, RSI *R. intestinalis*, SBV *S. variabile*.

The human gut microbiome consists of hundreds of species, several of which perform similar functional roles like starch degradation and butyrate production. Multiple species compete for substrates at each of the trophic levels, for example, several species can utilize starch (primary degraders), simple sugars (secondary consumers), and lactate (tertiary consumers) (Fig. 1). In order to mimic metabolic competition and cross-feeding interactions in-vitro, we aimed to assemble a multi-substrate and multi-species minimal microbiome. Firstly, we chose to reconstruct the metabolic interactions that lead to production of butyrate and propionate from multiple dietary fibres of general interest in the human gut, i.e. nonstarch polysaccharides (pectin and xylan), starch, inulin (potential prebiotic) and cellobiose (hydrolytic product of cellulose degradation)^10,26–28^. Next, we screened publicly available gut metagenomes to identify core species shared by a high proportion of the population. From the identified core species, we aimed to select species that are known to degrade either one or more of the chosen dietary fibres (pectin, xylan, starch, inulin, cellobiose), their breakdown products (glucose, fructose, xylose and galacturonate) or fermentation products (lactate and acetate). The specific strain selection was based on available information or genomic evidence for a given metabolic function of interest. The strains selected display at least one the following features: a) ability to utilize one or more of the dietary fibres; b) ability to utilize one or more of the simpler sugars released after breakdown of dietary fibres; c) ability to utilize one or more of the fermentation products formed by the action of other species in the community. We selected the strains in such a way that for each substrate there were at least two strains that could compete. As an example, *Bacteroides ovatus* and *Agathobacter rectalis* can both utilize starch (Fig. 1)^29,30^. To avoid selecting only the most abundant species, we decided to include species that are highly prevalent with high relative abundance and those that are highly prevalent but occur at low relative abundance. It is important to highlight that there are strains that likely meet the above mentioned criteria and could be used for similar design but different composition. However, for our study, we chose some well-studied strains (e.g. *Faecalibacterium prausnitzii*) and some less well-studied strains (e.g. *Lachnospiraceae* sp. 7_1_58FAA).

### Screening for Db-MM strains and their functional coverage in human gut microbiomes

**Fig. 2:**
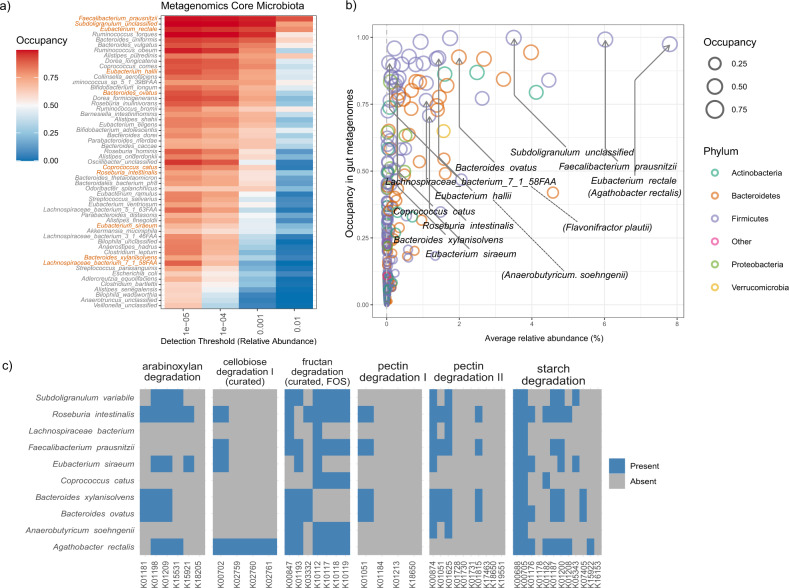
Core microbiota and features of Db-MM species. **a** Core microbiota occupancy in at least 50% of the samples at a minimum relative abundance of 0.0001 (Number of samples; *n* = 1144). **b** Occupancy-abundance relationship of Db-MM species. **c** Genomic potential based on gut metabolic modules for poly-, oligo and disaccharide degradation of selected Db-MM strains.

To identify core species, metagenomic data from 1144 stool samples of healthy individuals from the USA, Canada, Sweden, Netherlands, Denmark, Spain, Italy, France and Germany were obtained and analysed^31–40^. A total of 1634 metagenomic strains were aggregated to 622 species level taxa. Out of these 622 species, 64 were identified as members of the core microbiota based on their occupancy in at least 50% of the subjects at a minimum relative abundance of 0.0001 (Fig. 2a). From these 64 species, we selected ten representative strains from the three predominant families *Bacteroidaceae*, *Ruminococcaceae* and *Lachnospiraceae and Eubacteriaceae* following the rationale described above. We selected species that were present in low relative abundance (left side of Fig. 2b) and high relative abundance (right side of Fig. 2b) but having >70% occupancy. We chose to include this feature because relative abundances may reflect different growth behaviours and energy investment in biomass of these species. The phylogenetic relationship based on 16 S rRNA gene of the ten selected strains is shown in supplementary Fig. 1a. Additionally, to confirm that these ten species can co-occur an a given gut microbiome, we checked for their co-occurrences (Supplementary Fig. 1b, c and d). We observed 36% of the gut metagenomes had all ten species and overall, 60% of the times these ten species were positively co-occurring in pairs. This demonstrates that the chosen ten species are likely natural associates in human gut microbiomes.

The occupancy (70–100%) and known metabolic properties of the ten selected bacterial strains in our Db-MM are given in Supplementary Table 2. Furthermore, relevant metabolic properties were predicted from the genomes of the individual strains in the Db-MM, including polysaccharide degradation potential (Fig. 2c), the ability to utilize polysaccharide breakdown products and fermentation metabolites, and butyrate and propionate production (Supplementary Fig. 2 and 3).

Among the selected bacterial strains, *A.rectalis* (previously *E.rectale*), *F.prausnitzii*, *B.ovatus*, *R.intestinalis*, *B.xylanisolvens* and *E.siraeum* are all capable of utilizing one or more complex carbohydrates and can compete for resources (Supplementary Table 2). The other three strains, *C.catus*, *Subdoligranulum* sp. and *Anaerobutyricum soehngenii* (previously *E. hallii*) are not known to degrade complex carbohydrates. However, *A.soehngenii* can convert both D- and L-isomers of lactate with acetate to butyrate, whereas *C. catus* is only capable of utilizing the L- form of lactate to produce propionate. No information was publicly available regarding metabolic capabilities of *Lachnospiraceae* bacterium 7_1_58FAA. This strain is closely related to *Flavonifractor plautii* (Average Nucleotide Identify 98%) and from here on we use *F.plautii* as a synonym (Supplementary Table 4). Strains of *F.plautii* are known for their ability to degrade flavonoids as well as to produce acetate and butyrate^41^. In addition, previous genomic studies have predicted *F.plautii* to produce butyrate from lysine similar to closely related *Intestinimonas* species^42,43^. Notably, the ten strains in the Db-MM consortium together covered 71.7% of intestinal core KEGG KOs (present at least once in 75% of the HMP gut metagenomes), further providing evidence that the Db-MM consortium represents core functionality present in the Western human gut microbiome.

### Monoculture growth behaviour of individual Db-MM strains

**Fig. 3:**
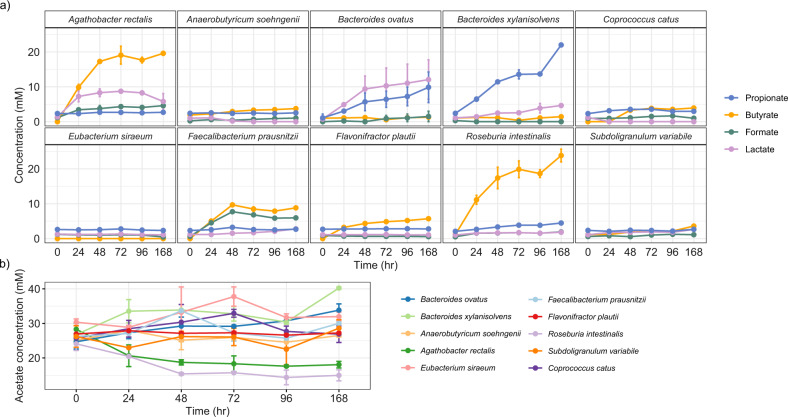
Metabolite analysis of Db-MM strains grown in monocultures. **a** Metabolites produced/consumed by Db-MM strains as monocultures grown in combination of xylan (beechwood), starch (from potato), inulin (from chicory), pectin (from apple), and cellobiose. **b** Changes in the concentration of acetate that was added at ~30 mM to support growth. Error bars indicate average measurements from duplicate cultures. The error bars represent standard deviation values for replicates at each time point.

Each strain was grown in duplicate as monoculture with a mixture of substrates (pectin, starch, inulin, xylan and cellobiose). After 24 h, *A.rectalis*, *F.prausnitzii*, *B.ovatus*, *R.intestinalis*, and *B.xylanisolvens*, grew substantially as revealed by an increase of >1.0 in OD_600_ and decline in pH, after 24 h along with the appearance of fermentation products, confirming their role as poly- and/or oligosaccharide degraders (Fig. 3a and Supplementary Fig. 4a-c). In contrast, weak or no growth was observed for *E.siraeum*, *F.plautii, C.catus*, *S.variabile* and *A.soehngenii*. Out of these five strains, *E.siraeum* is known to degrade complex polysaccharides (Supplementary Table 2). Among the butyrate producers, *R. intestinalis* produced the highest amount of butyrate, followed by *A.rectalis* and *F.prausnitzii* (Fig. 3a). The highest propionate concentrations were produced by *B.xylanisolvens* followed by *B.ovatus*. Lactate production was detected in the cultures of *B.ovatus*, *A.rectalis* and *B.xylanisolvens*. Since *A.rectalis*, *R. intestinalis*, and *A.soehngenii* grew well in the presence of acetate, the medium was supplemented with ~30 mM of acetate in all experiments^44–46^. Net production of acetate was observed for *E.siraeum*, *B*.*xylanisolvens* and *B.ovatus* (Fig. 3b).

### Strain composition and metabolites produced by Db-MM

**Fig. 4:**
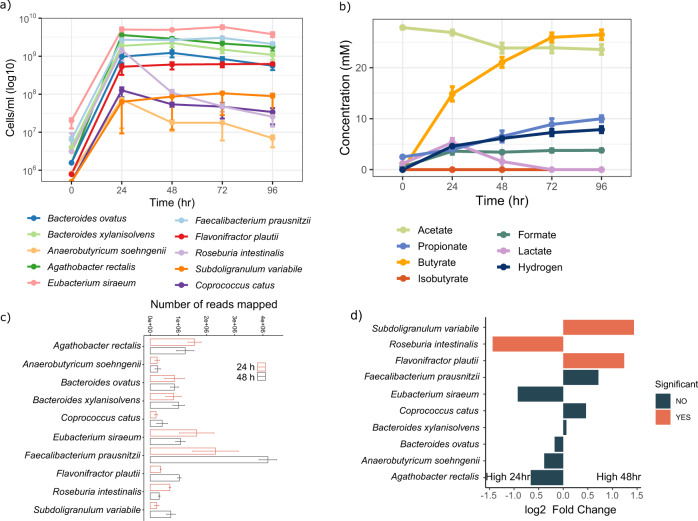
Metabolite and compositional dynamics of Db-MM. **a** Abundance of strains at different timepoints in Db-MM (*n* = 4). **b** Fermentation products observed in Db-MM (*n* = 4). **c** Number of metatranscriptome reads mapped to each of the ten Db-MM strains at 24 h and 48 h (*n* = 3). **d** Comparison of log2-fold change in number of metatranscriptome reads mapped to each of the ten Db-MM strains at 24 h and 48 h (*n* = 3). The error bars in panel a, b and c represent standard deviation values for replicates.

Absolute abundances of individual strains in the Db-MM consortium were determined using a combination of 16S rRNA gene amplicon sequencing and qPCR. A 50 to 5000-fold increase in 16S rRNA gene abundance of all ten strains at 24 h was observed indicating growth and coexistence (Fig. 4a). In the inoculum, uneven abundances were observed likely due to technical reasons, with *E.siraeum* being present at particularly high abundance compared to others (Supplementary Fig. 5a). *E.siraeum* was the most predominant strain (>10^9^ cells) at all timepoints followed by *A.rectalis*, *F.prausnitzii* and *B.xylanisolvens* at 24 h. The abundance of *R.intestinalis*, *C.catus* and *A.soehngenii* declined after 24 h while the remaining strains showed stable abundance until 96 h (Fig. 4a). We also observed decline in optical density after 24 h in line with the observed decline in some species (Supplementary Fig. 5b). As a community, DB-MM produced 26.5 ± 1.89 mM of butyrate after 96 h (Fig. 4b). This high concentration of butyrate was reproducibly observed in all four replicates and was higher than the amount produced by individual butyrate producers in monoculture from these substrates after this incubation period (Figs. 4b and 3a). Of note here is the increase in butyrate production to 23.82 ± 2.59 mM by *R.intestinalis* in monoculture at 168 h, indicating that prolonged incubation revealed the full butyrogenic potential of this strain. In contrast, the propionate concentration (9.97 ± 1.18 mM) produced by the Db-MM was lower than the maximal production of 13.67 ± 0.24 mM by *B. xylanisolvens* in monoculture at 96 h (Figs. 4b and 3a). The production of fermentation end products led to decline in the pH (Supplementary Fig. 5c). Lactate was produced by the Db-MM at 24 h, but was consumed at 48 h with a concomitant decrease in acetate concentration (Fig. 4b). Both D- and L-lactate were detected at 24 h, of which L-lactate was the abundant form (Supplementary Table 5). D,L-lactate was not detected at 72 h or 96 h, and the acetate concentration was stable after 48 h. *A.soehngenii*, *C.catus*, *F.plautii*, *S.variabile* and *E.siraeum* grew in the Db-MM as seen from increase in their 16S rRNA gene abundances (Supplementary Fig. 4a, Fig. 4a), indicating the presence of multi-species cross-feeding mechanisms supporting growth of all ten species.

### Metatranscriptomics reveals a community-level metabolic interaction network leading to the major SCFAs

To assess the transcriptionally active metabolism in the Db-MM, metatranscriptomic analysis was done for randomly selected three of the quadruplicate cultures at 24 h and 48 h of incubation. The transcripts were mapped to the genomes of the Db-MM strains for identifying strain specific transcriptional activity. Three strains, *F.prausnitzii*, *E.siraeum* and *A.rectalis*, showed the highest number of transcripts in line with their high abundance (Fig. 4c, Supplementary Table 6). At 48 h, *F.prausnitzii*, *C.catus*, *F.plautii*, and *S.variabile* had higher numbers of transcripts compared to 24 h, with the latter two strains showing a significant increase (log2-fold change >1.2, *p.adj* < 0.001; Fig. 4d; Supplementary Table 7), suggesting growth conditions favouring these strains at this time point. The number of transcripts for *R.intestinalis* were higher at 24 h compared to 48 h which is in concordance with the sharp decline observed in its abundance at 48 h (Fig. 4a). Overall, all the ten strains showed transcriptional activity demonstrating that these strains were actively co-existing in the consortia.

For an overarching view of the active metabolic pathways enriched in the Db-MM, pathway analysis was done using the community-level gene expression data. Gene-Concept network analysis revealed active pathways involved in polysaccharide breakdown and a concomitant activity of the pathways for the metabolism of breakdown products, such as glycolysis, fructose and mannose metabolism, and pentose phosphate pathway (Supplementary Fig. 6 and Supplementary Table 8). The functional analysis demonstrated interactions between the key pathways i.e., carbon metabolism, starch degradation, glycolysis/gluconeogenesis, pyruvate metabolism, pentose phosphate pathway, citrate cycle, butyrate and propionate metabolism (Supplementary Fig. 6 and Supplementary Table 8). In addition to the central metabolic pathways, an enrichment of transcribed genes involved in bacterial chemotaxis, flagellar assembly, two-component systems and vancomycin resistance was observed.

Reconstruction of metabolic pathways for butyrate and propionate production based on community gene expression data indicated that for butyrogenesis, both routes of the acetyl-CoA pathway, i.e., from butyrate kinase and butyrate-acetoacetate CoA-transferase, were active (Supplementary Fig. 7a). Notably, the expression of genes encoding pyruvate ferredoxin oxidoreductase (E.C:1.2.7.1) that converts pyruvate to acetyl-CoA was significantly higher at 48 h compared to 24 h (Supplementary Fig. 7a). In addition, the expression of genes associated with the conversion of the amino acid glutamate to butyrate was observed mainly at 48 h. Propiogenesis was observed *via* methylmalonyl CoA, acrylate and 1,2-PD pathways (Supplementary Fig. 7b). Genes coding for the key enzyme for the methylmalonyl CoA pathway, methylmalonyl-CoA mutase (EC:5.4.99.2), had higher expression at 48 h compared to 24 h, which coincided with the increase in propionate production (Fig. 4b; Supplementary Fig. 7b).

### Species specific transcriptional activity unravels active cross-feeding

To identify the nature of interactions between the ten strains, we applied a reverse ecology framework and compared complementarity/co-operation and competition between the Db-MM strains based on KEGG orthologs (KOs) present in genomes and actively expressed KEGG orthologs (KOs). By definition, the complementarity and competition index values range between 0–1, where a value closer to 1 indicates either higher co-operation or competition, respectively. While genome based calculation of competition index indicated high competition between *B.ovatus* and *B.xylanisolvens* (value of 1), gene expression based calculation indicated lower competition (*B.ovatus*-*B.xylanisolvens* = 0.87 and *B.xylanisolvens-B.ovatus* = 0.89) (Supplementary Fig. 8a, b). Genome-based calculation indicated no complementarity between either *Bacteroides* pairs while, gene expression based calculation of complementarity index revealed some complementarity between *B.xylanisolvens and B.ovatus* (0.1) but not between *B.ovatus*-*B.xylanisolvens* (0.0). (Supplementary Fig. 8c, d). Minor to no differences in competition and complementarity was observed for other strains which are phylogenetically more unrelated and belong to different genera. Notably, *F.prausnitzii* had lower competition predicted from gene expression data with *S. variabile* and *R. intestinalis* compared to genome-based prediction. Based on genome, *F.prausnitzii* had higher complementarity with the two *Bacteroides* strains (0.167), while based on gene expression data, it had 0.17 with *B.ovatus* and 0.08 with *B.xylanisolvens*. Based on genome, *A.soehngenii* had complementarity of 0.2 with the *B.xylanisolvens* strains but based on transcriptomics it was 0.114. These results highlight the need for caution when predicting pairwise interactions solely based on genomic content and the need for taking into account functional measurements such as metatranscriptomic or metaproteomes.

**Fig. 5:**
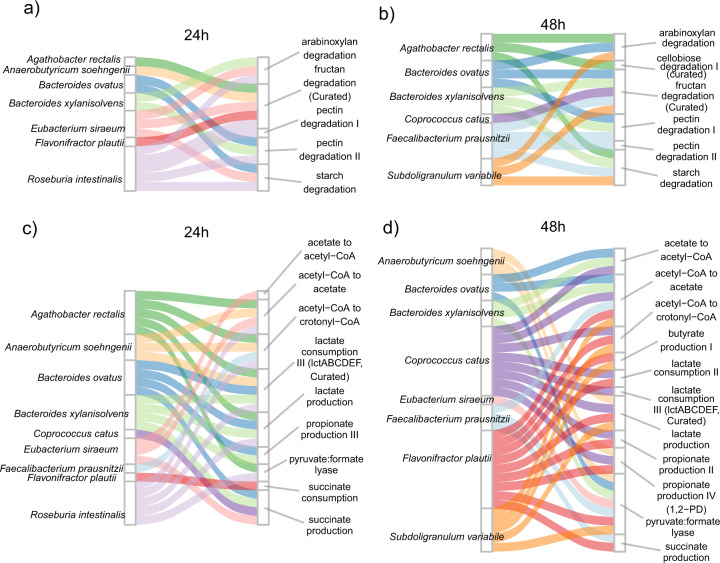
Species-function linkage based on transcriptional activity. Species showing active transcription of carbohydrate degradation modules at **a** 24 h and **b** 48 h. Species showing active transcription of volatile fatty acid metabolism at **c** 24 h and **d** 48 h.

For disentangling active metabolic processes at the strain-level, transcriptomic profiles were analysed using the gut metabolic modules (GMMs) framework. The GMMs reported previously were curated to include modules based on physiological information of the ten Db-MM strains (see methods for curation steps)^11^. Differences with respect to active expression of metabolic modules in the different strains were observed between the 24 h and 48 h samples (Fig. 5a, b). At 24 h, *R. intestinalis*, *B.ovatus, B.xylanisolvens, E.siraeum*, and *A.rectalis* were actively involved in expressing modules for fibre degradation. Conversely, at 48 h, *R. intestinalis* did not show any expression for modules for fibre degradation or organic acid metabolism (Fig. 5b). Both *C.catus* and *S.variabile* showed gene expression for fructan degradation, and *S.variabile* also showed gene expression starch and arabinoxylan degradation modules (Fig. 5b). For organic acid metabolism, at 24 h, expression of genes for acetate, lactate, butyrate and propionate production modules was dominated by *R.intestinalis*, *B.ovatus, B.xylanisolvens, E.siraeum*, and *A.rectalis* (Fig. 5c). At 48 h, *S.variabile*, *F.plautii* and *C.catus* showed higher expression of genes for organic acid metabolism modules (Fig. 5d). These transitions in transcribed modules were likely driven by public goods and metabolic end products that were released by the primary degraders.

### Species function relationships *via* gut metabolic modules

#### Carbohydrate degradation and utilization of public goods

In order to identify strain-specific activity with respect to polysaccharide degradation and simple sugar utilization in the Db-MM, we compared abundances of transcripts of corresponding GMM modules as well as individual KOs. Below we provide detailed strain-specific observations for each of the complex substrates and SCFA related metabolism.

#### Xylan utilization

**Fig. 6:**
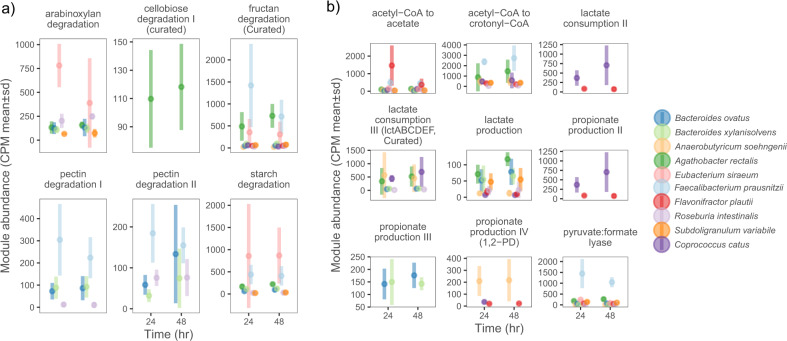
Comparison of gut metabolic modules (GMMs). **a** GMMs for poly-, oligo and di- saccharide degradation; **b** GMMs for short chain fatty acid production. Conversion of acetyl-CoA to crotonyl CoA is considered as proxy for butyrate production due to the difficulty in annotation of butyryl-CoA dehydrogenase using KEGG orthologues. Note that different scales were used for the y-axis values in each panel to account for different expression levels of each of the modules. The points represent the mean counts per million (CPM) values and bars represent standard deviation values for each of the replicate.

Comparison of abundances of GMMs for arabinoxylan degradation indicated high activity of *E.siraeum* (Fig. 6a). This observation is in line with a previous study demonstrating the ability of *E.siraeum* to degrade wheat bran, which also consists of xylan^47^. High abundance of *E.siraeum* transcripts encoding alpha−L − arabinofuranosidase (*abfA*; EC:3.2.1.55) and arabinoxylan arabinofuranohydrolase (*xynD*; EC:3.2.1.55, K15921) was detected at 24 h (Supplementary Fig. 9). Another known xylan degrader, *R. intestinalis*, showed high expression of genes encoding endo−1,4−beta−xylanase (*xynA*; EC:3.2.1.8) and xylan 1,4−beta−xylosidase (*xynB*; EC:3.2.1.37), which were shown to be important for degradation of xylan and resulting xylo-oligosaccharides^48^. Additionally, the gene encoding oligosaccharide reducing−end xylanase (*rexA*; EC:3.2.1.156) was expressed by *A.rectalis*, *R.intestinalis* and *S.variabile* indicating interactions driven by availability of public goods (Supplementary Fig. 9). Previously, *A.rectalis* was reported to utilize the hydrolysis products of arabinoxylan i.e., whole (A)XOS while releasing arabinose and xylose outside the cell, likely supporting xylose and arabinose-utilizing bacteria^49^. *E.siraeum* dominated the xylose degradation module, followed by *S.variabile* while relatively lower abundances of transcripts were observed for *A.rectalis*, *A.soehngenii*, *B.ovatus*, *R.intestinalis* (Supplementary Fig. 10). The arabinose that was released by the likely action of arabinofuranosidases produced by *E.siraeum* and *A.rectalis*, was converted by enzymes encoded by genes of the arabinose degradation module in several Db-MM members, including *A.soehngenii*, *B.ovatus*, *B.xylanisolvens*, *C.catus, R.intestinalis* and *S.variabile*.

#### Cellobiose utilization

The GMM for cellobiose degradation, which included the key genes for cellobiose phosphorylase and the cellobiose transporter, were only observed in *A.rectalis* (Fig. 6a). Investigation of the KEGG KO abundances within this module revealed expression of cellobiose phosphorylase genes in *E.siraeum*, *F.prausnitzii*, *R. intestinalis* and *S.variabile*. All the four strains are known for their ability to utilize cellobiose^46,50,51^. However, only *A.rectalis* showed high expression of the genes for the cellobiose-specific phosphotransferase system (PTS) transporters (PTS-Cel-EIIA, *celC*, *chbA*; PTS system, cellobiose-specific IIA component, PTS-Cel-EIIB, *celA*, *chbB*; PTS system, cellobiose-specific IIB component, and the PTS-Cel-EIIC, *celB*, *chbC*; PTS system, cellobiose-specific IIC component). These observations suggest that *A.rectalis* responded to the presence of cellobiose by expressing high amounts of specific PTS transporters.

#### Inulin utilization

Expression of the GMM for inulin (fructan) degradation was predominantly observed in *F.prausnitzii* (Fig. 6a). Inulin is a polysaccharide composed of fructose and a terminal glucose molecule. *F.prausnitzii* had high expression of genes encoding beta−fructofuranosidase (*sacA*; EC:3.2.1.26) and fructokinase (*scrK*; EC:2.7.1.4) demonstrating its unique niche for inulin within the Db-MM (Supplementary Fig. 11). The fructose degradation module was dominated by *A.rectalis*, *E.siraeum* and *F.prausnitzii* at 24 h, whereas at 48 h, *A.rectalis* and *F.prausnitzii* were most dominant (Supplementary Fig. 10).

#### Starch utilization

The GMM for starch degradation was dominated by *E.siraeum* and to some extent by *F.prausnitzii* (Fig. 6a). Starch degradation has previously been identified as a key role for *Bacteroides* species, including *B.ovatus*^52^. *E.siraeum* showed high expression of genes encoding enzymes involved in starch degradation such as alpha-amylase (*amyA, malS*; EC:3.2.1.1), 4−alpha−glucanotransferase (*malQ*; EC:2.4.1.25), pullulanase (*pulA*; EC:3.2.1.41), and maltose alpha−D − glucosyltransferase/alpha−amylase (*treS*; EC:5.4.99.16/3.2.1.1) (Supplementary Fig. 12). Investigation of the gene context revealed that the highly expressed amylase and pullulanase genes are co-localized with genes coding for an HTH-transcriptional repressor (*cytR*), maltose transporter (*malGF*), maltodextrin phosphorylase and 4-alpha-glucanotransferase (EC: 2.4.1.25). The combined action of 4-alpha-glucanotransferase and maltodextrin phosphorylase is suggested to be energy efficient^53^. Mining the genomes revealed the absence of a maltodextrin phosphorylase gene in genomes of the two *Bacteroides* strains used here, whereas this gene was present in genomes of *A.rectalis*, *F.prausnitzii*, and *S.variabile*. The high expression of the genes encoding amylolytic enzymes co-localized with *malGF* in *E.siraeum* indicates this organism’s transcriptional adaptation for utilizing amylose/maltodextrins and its capacity to compete with other bacteria capable of utilizing starch. Further investigations of the biochemical properties and substrate specificities of the amylolytic enzymes encoded by the *malGF* gene cluster will be required to understand their significance for the fitness of *E.siraeum* in presence of competition.

#### Pectin utilization

Among the five substrates used in this study, pectin is structurally the most complex, followed by xylan, inulin and starch and finally the disaccharide cellobiose^54^. Expression of pectin degradation modules was dominated by *F.prausnitzii*, *B.ovatus* and *B.xylanisolvens* (Fig. 6a). Pectinesterase (EC 3.1.1.11) is an important enzyme that produces de-esterified substrates for polygalacturonases and lyases^55^. In the Db-MM, *F prausnitzii*, showed high gene expression for pectinesterase at 24 h (Supplementary Fig. 13). *B.ovatus* dominated expression of the gene encoding 2-dehydro-3-deoxyphosphogluconate aldolase/(4 S)-4-hydroxy-2-oxoglutarate aldolase (*eda*; EC:4.1.2.14/4.1.3.42) followed by *R.intestinalis*, *F.prausnitzii* and *B.xylanisolvens*. Similar trends were observed for expression of the gene coding for 2-dehydro-3-deoxygluconokinase (*kdgK*; EC:2.7.1.45). The gene encoding 4-deoxy-L-threo-5-hexosulose-uronate ketol-isomerase (*kduI*; EC:5.3.1.17), involved in degradation of polygalacturonate was predominantly expressed by *B.ovatus* and *B.xylanisolvens* at 24 h, whereas at 48 h the highest *kduI* expression was found in *F.prausnitzii* (Supplementary Fig. 13). Thus, in the Db-MM, the majority of pectinolytic activity was observed in *F.prausnitzii, B.ovatus* and *B.xylanisolvens*. The major component of pectin is galacturonate, and the genes involved in its degradation were highly expressed in *F. prausnitzii* at 24 h, and to a lower extent in *B.ovatus* and *B.xylanisolvens*, further supporting the role of *Bacteroides* spp. in pectin utilization (Supplementary Fig. 10).

Together, these observations of gene expression for carbohydrate (polysaccharides and subsequent public good) degradation modules demonstrated differences in the inter-species response to available substrates.

#### SCFA producers and cross-feeding on excreted metabolites

From the SCFA analysis and transcriptomics based pathway reconstruction, we observed an active expression of modules involved in butyrate and propionate metabolism (Fig. 4b, Supplementary Fig. 7). To link the metabolic processes involved in SCFA production to DB-MM strains, we compared the abundance of the expressed metabolic modules. In monoculture, the highest formate production was observed in *F.prausnitzii*, and in the Db-MM metatranscriptome, high expression of the pyruvate:formate lyase encoding gene of *F.prausnitzii* was detected (Fig. 6b). This suggests that the majority of the formate in the Db-MM was produced by *F.prausnitzii*. Propionate formation in monoculture was observed for *B.ovatus* and *B.xylanisolvens*, which both employ the succinate pathway *via* methylmalonyl-CoA^27,56,57^. However, the amount of propionate produced in consortia was ~10 mM less than that observed in monoculture of *B.xylanisolvens*. In the Db-MM, we observed active expression of the GMM ‘propionate production III’ in both *Bacteroides* strains (Fig. 6b). Two other GMMs for propionate production (II and IV) were found to be active in *C.catus* and *A.soehngenii*, respectively. The propionate production module II occurs via the consumption of lactate and is active in *C. catus*, while propionate production IV proceeds *via* the 1,2-PD pathway, which is active in *A.soehngenii*. D-lactate is utilized by *C.catus*, whereas *A.soehngenii* can utilize both D- and L-lactate^44,56,58^. In the Db-MM, we incorporated the potential for this cross-feeding by including multiple species (*A.rectalis*, *B.ovatus* and *B.xylanisolvens*) capable of producing lactate and multiple species capable of utilizing lactate and acetate to produce either butyrate (by *A.soehngenii*) or propionate (*C.catus*). Gene expression analysis and the disappearance of lactate in consortia at 48 h supports the active cross-feeding between lactate producers and consumers (Fig. 6b). In a previous study on physiological properties of *A.soehngenii*, we reported its ability to utilize several mono- and disaccharides including glucose, fructose, galactose and maltose^59^, which are likely available as public goods in cultures of the Db-MM consortium. Interestingly, a previous study reported that in monocultures, glucose was utilized prior to lactate by *A.soehngenii*^44^. Recently, we identified a unique gene cluster, *lctACDEFG*, that is highly conserved in *Anaerobutyricum* and *Anaerostipes* species and that enables growth on low-energy D-,L-lactate^60^. We identified high expression of the *lctACDEFG* gene cluster encoded by *A.soehngenii* in the Db-MM. In addition, *Anaerobutyricum* species have the ability to utilize 1,2-PD and thereby contribute to propionate formation^61^. Our present observations indicate the existence of a high competition potential for lactate in the Db-MM. The ability of *A.soehngenii* to utilize the D-,L- form of lactate can be advantageous. In addition, *A.soehngenii* likely plays a key role in the conversion of 1,2-PD to propionate. Using an enzymatic assay, we could confirm the presence of both D- and L-lactate in cell-free supernatants at 24 h. These observations provide crucial insights into the importance of micro-niche specialization (such as the ability to utilize L- or D-lactate) as an important process for coexistence.

For identifying active genes involved in butyrate formation, we used the acetyl-CoA to crotonyl-CoA GMM as a proxy instead of the pre-defined butyrate production I and II GMMs^11^. This was mainly due to the lack of confidence in assignment of genes involved in the conversion of crotonyl-CoA to butyryl-CoA to butyrate by KEGG KO families. *F.prausnitzii*, *A.rectalis*, *A.soehngenii, S.variabile* and *C. catus* were among the most active contributors to the transcripts of genes of the acetyl-CoA to crotonyl metabolic module (Fig. 6b). In the Db-MM, the final step proceeds *via* two different pathways, one *via* the butyryl-CoA:acetate CoA-transferase (*A.soehngenii*, *A.rectalis*, *F.prausnitzii*, *F.plautii* and *R. intestinalis*), which is the major contributor, and minor contribution of the second pathway *via* butyrate kinase (*S.variabile*).

### Comparison of amino acid biosynthesis and degradation modules suggests potential cross-feeding

In addition to butyrogenesis from sugars, we also observed active gene expression related to butyrate formation *via* lysine. Recently identified genes involved in butyrogenesis from lysine in *F.plautii* were actively expressed, which prompted us to further investigate amino acid biosynthesis and degradation modules (Supplementary Fig. 14)^42^. Since the growth medium included undefined substrates such as yeast extract and casitone, these amino acids could well have been a source of energy for some bacteria in our experiments. These medium components were incorporated because the human gut is widely accepted to be a nutritionally rich ecosystem. However, competition for amino acids due to similar auxotrophies cannot be ruled out. Biosynthesis pathways for the majority of the amino acids were dominated by *F.prausnitzii*, *E.siraeum* and *A.rectalis* (Supplementary Fig. 15). The three lysine biosynthesis modules were dominated by *F.prausnitzii* and *E.siraeum* whereas the butyrogenic lysine degradation module was active only in *F.plautii* (Supplementary Fig. 15 and 16). Additionally, *F.plautii* had active GMMs for isoleucine, leucine and tryptophan degradation (Supplementary Fig. 16). These observations indicate the potential role of *F.plautii* in amino acid degradation.

## Discussion

Minimal microbiomes provide a promising avenue for improving our knowledge of interspecies interactions^25^. In complex natural ecosystems, metabolic behaviours and interspecies interactions are highly dynamic and dependent on factors like available resources and other species in the community. Therefore, to investigate interspecies interactions that mimic natural systems, as much as possible, multi-species minimal microbiomes with multiple substrates can to be constructed and investigated. In this proof of concept study, we demonstrate the feasibility of rationally designing such a multi-species minimal microbiomes with multiple substrates and investigating metabolic interactions using metatranscriptomics.

The Db-MM consists of species that are representatives of the core microbiota, are likely natural associates and cover ~70% of the functions (KEGG KOs) present in westernized human gut microbiomes. The growth of non-polysaccharide/oligo-saccharide degrading bacteria such as *A.soehngenii*, *C.catus*, and *S.variabile* in consortia indicated the coexistence of Db-MM species is driven by cross-feeding interactions. Since all the Db-MM strains have known genome sequences (nine reported previously and *C.catus* in this study) we were able to investigate species-specific transcriptomic profiles using metatranscriptomics. We identified species involved in breakdown of complex substrates and consumption of liberated simpler sugars. Furthermore, we observed that cross-feeding occurred not only for simpler sugars but also for fermentation end products as observed by the consumption of D,L-Lactate at 24 h.

Notable observations were made in our study regarding the core polysaccharide degrading species. The transcriptional activity of *E.siraeum* was mostly specific for xylan and starch degradation which was of interest since the Db-MM included well-studied core bacteria such as *R. intestinalis* (key degrader of xylan), *Bacteroides* species and *A.rectalis* (starch degraders)^10,17,48,62^. Further co-culture experiments of *E.siraeum* with these and selected other species of the Db-MM are required to better understand the competitive behaviour of *E.siraeum*. As expected, known polysaccharide degraders showed transcriptional activity related to fibre degradation. The activity of fibre degraders like *E.siraeum*, *F.prausnitzii*, *B.ovatus*. *B.xylanisolvens*, *A.rectalis* and *R.intestinalis* contributed to creation of resources (simpler sugars and fermentation end products) leading to increase in the availability of niches for non-polysaccharide degraders like *S.variabile*, *C.catus*, *A.soehngenii* and *F.plautii*. These observations exemplify the trophic interactions between polysaccharide degrading and non-polysaccharide degrading species isolated from the human gut.

An important cross-feeding interaction observed in the human gut is conversion of lactate to butyrate and/or propionate. Of interest were the butyrate and propionate-producing *A.soehngenii, C.catus* and *F.plautii*. D,L-lactate was produced by *A.rectalis* and *B.ovatus*, both of which reached high abundances along with other carbohydrate-utilizing bacteria. The observed low abundance of *A.soehngenii* suggest that this organism likely follows a K-strategy to achieve coexistence as a member of the core microbiota in the human gut. The high expression of the GMM for lactate consumption by *C.catus* suggests that this organism likely competes with *A.soehngenii* for L-lactate and co-culture competition experiments can be designed in future to study this in detail.

While this proof-of-concept study provides crucial insights into interspecies metabolic interactions we would like to note some key aspects for future studies. The replicates we used are technical as we used the same inoculum mixture for all replicates. This approach allowed us to limit technical variation. When scaling-up complexity, especially with closely related strains, either full-length 16S rRNA gene sequencing or metagenomic approaches need to be considered. We used a batch culturing approach, and future studies using in-vitro or in-vivo models need to incorporate denser longitudinal measurements with the goal to study metabolic interactions and community dynamics. We measured composition, metabolites and metatranscriptomes at 24 h intervals. It is possible that key interactions may take place within the first 24 h of assembly as we observed the majority of the strains reaching stationary phase at 24 h in our study. Moreover, combining our approach with stable-isotope probing (SIP) by labelling complex substrates, it may be possible to track nutrient utilization dynamics^63^. Additionally, the current Db-MM can be expanded in future to incorporate additional trophic interactions such as those involving methanogenic, sulfidogenic and/or autotrophic (H_2_/CO_2_) metabolism to identify the potential role of thermodynamic constraints exerted by accumulation of H_2_, a by-product of fermentation as observed in our study on driving syntrophic interactions. Nevertheless, the primary goal of our study was to demonstrate the feasibility to identify interspecies interactions and metabolic roles of core bacteria in a competitive consortium and was achieved by using the current experimental approach.

In conclusion, we reconstructed the major metabolic pathways involved in conversion of dietary fibres, as well as amino acids such as lysine to SCFAs using only ten core gut bacteria. We demonstrate the feasibility of identifying previously overlooked metabolic behaviours of core bacteria, as well as interspecies metabolic interactions and unique metabolic niches such as growth on low-energy substrates. The findings of this study can help in future studies aimed towards building predictive metabolic interaction models of the gut microbiome.

## Materials and methods

### Metagenomic screening

Taxonomic composition data from metagenomic studies was obtained from the curatedMetagenomicData data package (Supplementary Table 1)^64^. These included a total of 1144 samples from Germany (*n* = 97), Denmark (*n* = 177), Spain (*n* = 71), France (*n* = 61), Italy (*n* = 11), The Netherlands (*n* = 471), Sweden (*n* = 70), United States of America (*n* = 194) and Canada (*n* = 3)^31–40^. Core microbiota analysis was done using the microbiome R package^65^. To identify co-occurrence patterns of the ten selected species, we converted the abundance into a presence/absence matrix. We used the probabilistic model of species co-occurrence available in the R package cooccur (v1.3)^66,67^. In order to identify what fraction of functions that are usually detected in human gut metagenomes are covered by the Db-MM consortium, we searched publicly available metagenomes in the Joint Genome Institute Integrated Microbial Genomes & Microbiome System (JGI-IMG/MER) database^68^. Hundred human gut metagenomes were randomly chosen from the human microbiome project, and their KEGG ortholog profiles were downloaded (available on GitHub; https://github.com/microsud/Db-MM-10)^69^. We first identified KOs that are present at least once in 75% of the samples. We then calculated the number of combined KOs that were identified in genomes of the Db-MM strains.

### Bacterial strains and growth conditions

The following strains were obtained from the Leibniz Institute DSMZ-German Collection of Microorganisms and Cell Cultures (Braunschweig, Germany): *Agathobacter rectalis* [previously named *Eubacterium rectale*] (DSM 17629), *Eubacterium siraeum* (DSM 15702), *Roseburia intestinalis* (DSM 14610), *Subdoligranulum variabile* (DSM 15176). *Anaerobutyricum soehngenii* [previously named *Eubacterium hallii*] (DSM 17630) was kindly provided by Prof. Harry J. Flint’s group (University of Aberdeen, UK). The strains selected from the human microbiome project (HMP) catalogue were *Bacteroides* sp. 3_1_23 (closest relative *Bacteroides ovatus*), *Bacteroides* sp. 2_1_22 (closest relative *B. xylanisolvens*) and *Lachnospiraceae* bacterium 7_1_58FAA (*F. plautii*). These HMP strains and *Faecalibacterium prausnitzii* (A2-165) were kindly provided by Dr. Clara Belzer (Laboratory of Microbiology, Wageningen University & Research, the Netherlands). *Coprococcus catus* (ATCC27761) was obtained from the American Type Culture Collection (ATCC). Bacterial cells were stored at −80°C in pre-reduced 20% polyethylene glycol in phosphate buffer saline (PBS). The following medium was used for all experiments containing, KH_2_PO_4_ (0.408 g/L), Na_2_HPO_4_.2H_2_O (0.534 g\L), NH_4_Cl (0.3 g/L), NaCl (0.3 g/L), MgCl_2_*6H_2_O (0.1 g/L), NaHCO_3_ (4 g/L), yeast extract (2 g), casitone (2 g), beef extract (2 g), sodium acetate (2.46 g), peptone (2 g), 1 ml of trace elements in acid (HCl, 50 mM; H_3_BO_3_, 1 mM; MnCl_2_.4H_2_O, 0.5 mM; FeCl_2_.4H_2_O, 7.5 mM; CoCl_2_.6H_2_O, 0.5 mM; NiCl_2_.6H_2_O, 0.1 mM; ZnCl_2_, 0.5 mM; CuCl_2_.2H_2_O, 0.1 mM), 1 ml of trace elements in alkaline (NaOH, 10 mM; Na_2_SeO_3_, 0.1 mM; Na_2_WO_4_.2H_2_O, 0.1 mM; Na_2_MoO_4_.2H_2_O, 0.1 mM), 0.5 g Cysteine-HCl, 1 mL Haemin solution (50 mg haemin; 1 mL NaOH, 1 N; 99 mL dH2O), and 0.2 mL Vitamin K1 solution (0.1 mL vitamin K1; 20 mL ethanol, 95%). The medium was boiled and cooled under N_2_ gas, and 45 ml aliquots were distributed in 120 ml serum bottles. The bottles were sealed with butyl rubber stoppers and crimped with aluminium caps, and the headspace gas was exchanged with an N_2_/CO_2_ gas mixture (4:1 ratio). After autoclaving and before inoculation, 1% vitamin in CaCl_2_ solution was added. The vitamin solution contained per litre: biotin (20 mg), nicotinamide (200 mg), p-aminobenzoic acid (100 mg), thiamine (B1, 200 mg), pantothenic acid (100 mg), pyridoxamine (500 mg), cyanocobalamin (B12, 100 mg), riboflavin (100 mg) dissolved in deionized water. For pre-cultures, the bacteria were grown on combinations of different carbon sources (Supplementary Table 1). A mixture of 1 g/L of xylan (beechwood, Apollo scientific, U.K), starch (from potato), inulin (from chicory), pectin (from apple), and cellobiose dissolved in anoxic water was added to the growth media. The pre-cultures were incubated nonshaking at 37 °C in the dark for 24 h.

### Growth in monoculture and in consortia

For testing growth of monocultures on a mixture of complex carbohydrates, 0.5 ml of actively growing pre-culture in midexponential phase was used to inoculate duplicate bottles containing 50 ml fresh medium. Growth (OD_600_), pH and SCFA production were measured every 24 h until 168 h. For testing growth of mixed cultures on a mixture of complex carbohydrates, the OD_600_ of the actively growing pure pre-cultures was measured, strains were combined at approximately equal optical densities (OD_600_ = 0.3), and used for inoculation of quadruplicate bottles (B1, B2, B3 and B4) containing 400 mL fresh medium amended with 1 g/L of each complex carbohydrate. Samples (2 ml) were taken for measuring growth (OD_600_), pH, SCFA production and DNA extraction for community analysis every 24 h until 168 h after inoculation. Samples for RNA extraction/metabolites (20 mL) were taken at 24 h and 48 h and were centrifuged at 4816× *g* for 30 min at 4 °C. After centrifugation, the pellets were snap frozen with liquid nitrogen, and immediately stored at −80°C until further processing.

### Metabolite analysis

Analyses of SCFAs (formate, acetate, propionate, iso-butyrate and butyrate), lactate and monosaccharides (glucose, xylose and fructose) were done using a Shimadzu Prominence-i LC-2030c HPLC liquid chromatograph equipped with a Shodex SUGAR SH1011 column. For the mobile phase, 0.01 N H_2_SO_4_ was used. Samples were prepared by centrifuging 1 mL of the bacterial culture at 12,000 × *g* for 10 min and adding 400 µL of the supernatant to 600 µL of crotonate (30 mM) used as an internal standard. Standard curves for SCFAs, lactate and monosaccharides were prepared using four different concentrations (2.5, 5, 10 and 20 mM). Hydrogen gas was measured by withdrawing 0.2 ml samples from the headspace of the culture bottles. A Compact GC 4.0 (Global Analyser Solutions, The Netherlands) with Molsieve 5 A column operated at 100 °C coupled to a Carboxen 1010 pre-column was used to measure H_2_. The standard curve was prepared from a bottle containing 2.5, 5 and 10% H_2_. An enzymatic assay was used for detecting D and L-isomers of lactate in the culture supernatant following the manufacturer’s instructions (Megazyme, Ireland).

#### *Coprococcus catus* genome sequencing

Genomic DNA of *Coprococcus catus* (ATCC^®^ 27761^™^) was extracted using the MasterPure Gram-positive DNA purification kit (Epicentre, Amsterdam). The quality of the extracted DNA was measured using a NanoDrop 2000 spectrophotometer (Thermo Scientific, USA). The genome was sequenced using Illumina NovaSeq-PE150 at Novogene (Novogene Europe Laboratory: Cambridge, UK). The forward and reverse reads were filtered using Trimmomatic (v0.36) (settings: ILLUMINACLIP:TruSeq3-PE.fa:2:30:10:2:keepBothReads LEADING:3 TRAILING:3 MINLEN:36)^70^. High-quality filtered forward and reverse reads were assembled into contigs using SPAdes (v3.5.0)^71^. Filtered reads were mapped to contigs using bwa (alignment *via* Burrows-Wheeler transformation, v0.7.5a-r405), and coverage statistics were calculated using samtools (v1.9-58)^72,73^. Contigs with less than 500 bp were removed, and remaining high-quality 67 contigs were annotated using Prokka (v1.12)^74^. The draft genome sequence of *Coprococcus catus* (ATCC 27761) has been deposited at GenBank/EMBL-EBI under the bioproject number PRJNA622412 with the accession number JAAXCM000000000 *Coprococcus catus* VPIC661.

#### DNA extraction and amplicon sequencing of consortia

Total DNA of the consortia was extracted using the DNeasy PowerSoil Kit (QIAGEN, Hilden, Germany) following the manufacturer’s instructions. The DNA concentration was measured using a Nanodrop spectrophotometer (NanoDrop Technologies, Wilmington, DE, USA). The hypervariable region V5-V6 (~280 bp) of the 16S ribosomal RNA (rRNA) gene was amplified with Phusion Hot start II DNA polymerase (2 U/μl) and 0.05 µM of each primer (784 F–1064 R) with sample-specific barcodes at their 5’-end. The amplification program for PCR included a 30 s initial denaturation step at 98 °C, followed by 25 cycles of denaturation at 98 °C for 10 s, annealing at 42 °C for 10 s, elongation at 72 °C for 10 s, and a final extension at 72 °C for 7 min. Purified PCR products were quantified using the Qubit dsDNA BR Assay Kit (Life Technologies, USA) and were pooled in equimolar amounts into one single library and sequenced on a NovaSeq 6000 2 × 150 bp paired-end mode at GATC Biotech (Konstanz, Germany; now part of Eurofins Genomics Germany GmbH).

### 16S rRNA gene-targeted quantification of total bacterial load

The extracted DNA from bacterial consortia was diluted to 15 ng/uL and qPCR measurements were performed in triplicate in 10-μL reactions in an iQ5 iCycler (Bio-Rad, Veenendaal, the Netherlands) using the iQ SYBR Green Supermix kit (Bio-Rad). Standard curves were obtained using serial dilutions of a known amount of plasmid DNA containing a fragment of the 16S rRNA gene of *Dehalococcoides mccartyi* CBDB1. The primers were 341 F 5’-CCTACGGGAGGCAGCAG-3’ and 534 R 5’-ATTACCGCGGCTGCTGGC-3’, and the thermal cycling conditions were 95 °C for 10 min, followed by 39 cycles of 95 °C for 15 s, 60 °C for 30 s, 72 °C for 30 s.

### Amplicon data processing and community analysis

The raw reads were processed in R using the DADA2 R package (v 1.20.0)^75^. After filtering the reads with low quality, removal of reads with more than 2 errors and those matching the PhiX (filterAndTrim function), and chimeric sequences (removeBimeraDenovo, consensus method), a total of 1,010,000 reads were obtained from 23 samples, including one mock community comprised of known 16S rRNA gene sequences of microorganisms occurring in the human gut, and two inoculum samples which contained each candidate strain mixed in equal cell densities^76^. The raw paired-end 16S rRNA gene amplicon sequences have been submitted to the European Nucleotide Archive (ENA; https://www.ebi.ac.uk/ena) under project accession number PRJEB36253.

The taxonomic assignment was done using a custom database containing the full-length 16S rRNA gene sequences for all of the ten strains used in the present study (database available at https://github.com/microsud/Db-MM-10)^77^. The classification was done using the RDP classifier^78^. Unclassified amplicon sequence variants (ASVs) were removed before further analyses. The ASVs identified by DADA2 were collapsed at species level and counts were corrected for the differences in 16S rRNA gene copy number identified in the individual strain genomes (Supplementary Table 2). This was done by dividing raw species level counts with copy number. The corrected counts were scaled to the total 16S rRNA gene copies per sample obtained from qPCR thus correcting for sequencing depth and obtaining quantitative microbiota profiles similar to a previous study based on cell-counts^79^. Due to the limited complexity and exact genome-based knowledge of 16S rRNA gene copy numbers for each strain (Supplementary Table 2), qPCR was preferred over fluorescence-activated cell sorting (FACS) which can lead to bias due to cell aggregation^80^. Further analysis of the community composition and structure was done using the microbiome R package (v 1.14.0)^65^. Data visualization was primarily done using ggplot2 (v 3.3.3) and ggpubr (v 0.4.0) R packages.

### RNA extraction and sequencing

For extraction of total community RNA, the TRIZOL^TM^ method was followed^81^. The snap frozen cell pellets were suspended in solution consisting of 4 µl of p-mercaptoenthanol, 0.4 ml of buffer RLT and 1 ml of Trizol® reagent. The suspended pellets were distributed in tubes containing 0.8 g of glass beads (diameter 0.1 mm) and subjected to bead beating for 1 min at 5.5 m/s with ice cooling steps in between for three times. Following the mechanical disruption, 0.2 ml of ice-cold chloroform was added. This solution was then gently mixed and then subjected to centrifugation at 12,000 × *g* for 15 min at 4 °C. Further processing to obtained RNA was done using the RNeasy Mini kit (Qiagen, USA). Genomic DNA digestion was done with an on-column DNase digestion step during RNA purification using the RNase-free DNase I recombinant enzyme (Roche Diagnostics, Germany). The isolated total RNA was stored at −80 °C. RNA concentrations were determined with a Nanodrop spectrophotometer (NanoDrop Technologies, Wilmington, DE, USA), and total RNA quality check was performed using the Bioptic Qsep100 (GC biotech, Waddinxveen, the Netherlands). The removal of rRNA and sequencing was done using Illumina NovaSeq-PE150 at Novogene (Novogene Europe Laboratory: Cambridge, UK). We analysed triplicates for (24 h and 48 h) RNA-seq data from B1, B2 and B3 replicate cultures. The raw paired-end sequences can be accessed via the European Nucleotide Archive (ENA; https://www.ebi.ac.uk/ena) under the project accession number PRJEB36253.

### RNA data processing and analysis

A total of 131,902,534 raw paired-end reads were obtained from six samples (triplicates for 24 h and 48 h) of consortia (Supplementary Table 3). We followed the approach described in the SAMSA2 pipeline for data processing^82^. The forward and reverse adaptors were filtered using Trimmomatic (v0.36) (settings: PE -phred33, SLIDINGWINDOW:4:15, MINLEN:70)^70^. High-quality filtered forward and reverse reads were merged using pear (v0.9.10)^83^. The ribosomal reads were removed with SortMeRNA (v2.1)^84^.

A custom database was created from genome sequences of all the bacterial strains used in this study. All the genome sequences were downloaded from the NCBI genome database, except for *C. catus* (ATCC^®^ 27761^™^) that was sequenced in this study (see above). The genomes of all ten strains were re-annotated using Prokka (v1.12) for the sake of consistency in gene annotations^74^. The 16S rRNA gene copy numbers for individual strains were identified using the barrnap (v0.9) tool^85^. The amino acid fasta sequences were concatenated into a single file and converted to a DIAMOND (v 0.9.22.123) compatible database using *makedb* function^86^.

After removal of ribosomal sequences, the remaining reads were annotated with DIAMOND using blastx. The top hits assigned to amino acid sequences of all ten strains (from Prokka analysis) were then used for downstream analysis in R. The corresponding codes are available at (https://github.com/microsud/Db-MM-10). For linking metatranscriptomics reads to KEGG databases, the concatenated amino acid fasta sequences database of all ten strains were submitted to GhostKola for KEGG ortholog (KO) annotations^87,88^. The locus tags of each genome were then linked to KO annotations for pathway analysis. Gene enrichment analysis was done using clusterProfiler (v 3.17.5)^89^. Pathway analysis was done using R/BioC package pathview (v 1.29.1). Differential abundance analysis was done using the R/Bioc package DESeq2 (v 1.29.16)^90^.

### In silico prediction of pairwise competition and complementarity

Using the KEGG ortholog (KO) annotations of metatranscriptomics data, a metabolic network was reconstructed. This metabolic network was used to identify a set of seed compounds, which are acquired by each bacterium from the extracellular environment and non-seed compounds, which are produced by the bacterium itself. Briefly, the KO annotations for each genome (or expressed transcriptome) is converted into a genome-scale composite metabolic network graph. The seed and non-seed compounds for each bacterium are identified and pairwise metabolic complementarity and competition were calculated in R using the RevEcoR package (v 0.99.3)^20,91^.

First, we calculated competition and complementarity indices using genome-based KO annotations. Next, we calculated competition and complementarity indices using only the KOs that that were expressed by each of the strains in consortia. In contrast to the previous study, we also used actively expressed genes instead of using genome-based KO annotations as input data. This allowed us to calculate “active” interspecies interactions.

### Gut metabolic modules (GMMs)

The GMM framework was used for linking species transcriptomes to metabolic modules^11,92^. We curated the metabolic modules based on prior information about the strains used in this study. We included additional modules for cellobiose degradation, lactate consumption (via bifurcation mechanism), 1,2- propanediol (1,2−PD) production (via two routes, I-lactaldehyde, II-hydroxyacetone), and propionate production IV (*via* 1,2−PD), and we curated the fructan degradation module for specificity. We added the amino acid biosynthesis module as per their respective KEGG modules and formatted it according to GMM rules for KO requirements such as tabs and commas. The curated GMMs are available at the GitHub repository of this study (https://github.com/microsud/Db-MM-10). The omixer-rpmR R package (v0.3.1) was used for profiling of metabolic modules^92^. Here, the input was KO count normalized for library size differences using the *cpm* function in edgeR R package (v 3.34.0)^93^. The parameters for the *rpm* function in omixer-rpmR were as follows, score.estimator = “median”, contribute = 0.5, KO = 2, distribute = NULL.

## Supplementary information

Supplementary Information

## Data Availability

Raw transcriptomics and 16S rRNA gene amplicon sequencing data are available under the BioProject PRJEB36253. The wet lab data including HPLC, O.D. and pH measurements are available here https://github.com/microsud/Db-MM-10/tree/master/data_raw/02_wetlab. The *Coprococcus catus* VPIC661 genome is deposited at EMBL/ENA with accession number JAAXCM000000000.

## Code availability

All processed data and custom codes used for analysis reported in the manuscript are available on GitHub: https://github.com/microsud/Db-MM-10 and archived at the zenodo repository^77^.
