# Author Correction: Inter-species metabolic interactions in an in-vitro minimal human gut microbiome of core bacteria

**DOI:** 10.1038/s41522-022-00339-3

**Published:** 2022-10-06

**Authors:** Sudarshan A. Shetty, Ben Kuipers, Siavash Atashgahi, Steven Aalvink, Hauke Smidt, Willem M. de Vos

**Affiliations:** 1 Laboratory of Microbiology, Wageningen University & Research, Wageningen, The Netherlands; 2 Department of Microbiology, Radboud University, Nijmegen, The Netherlands; 3 Human Microbiome Research Program, Faculty of Medicine, University of Helsinki, Helsinki, Finland; 4 Present Address: Department of Medical Microbiology and Infection prevention, Virology and Immunology Research Group, University Medical Center Groningen, Groningen, The Netherlands

**Keywords:** Microbial ecology, Microbiome

Correction to: *npj Biofilms and Microbiomes* 10.1038/s41522-022-00275-2, published online 08 April 2022.

In the Methods of the original Article, strain accession number DSM 1736 was displayed instead of DSM 17630, and errors were introduced in the composition of the growing medium. These errors have now been corrected in both the HTML and PDF versions of this Article. The Supplementary Information file has also been updated.

## Supplementary information

Supplementary Information

