# Supplementary material for: Inter-species Metabolic Interactions in an In-vitro Minimal Human Gut Microbiome of Core Bacteria
Source: NPJ Biofilms Microbiomes. 2022 Apr 8;8:21. doi: 10.1038/s41522-022-00275-2 (PMC8993927; doi:10.1038/s41522-022-00275-2)
Supplement: Supplementary file 1 — Supplementary Information [file 41522_2022_275_MOESM1_ESM.pdf]

Inter-species Metabolic Interactions in a an *In-vitro* Minimal Human Gut Microbiome of Core Bacteria

Sudarshan A. Shetty[1,#] , Ben Kuipers[1], Siavash Atashgahi[1,2], Steven Aalvink[1], Hauke Smidt[1], Willem M. de Vos[1,3]*

[1]Laboratory of Microbiology, Wageningen University & Research, Wageningen, The Netherlands.

[2]Department of Microbiology, Radboud University, Nijmegen, The Netherlands.

[3]Human Microbiome Research Program, Faculty of Medicine, University of Helsinki, Helsinki, Finland.

[#]Present address: Department of Medical Microbiology and Infection prevention, Virology and Immunology Research Group, University Medical Center Groningen, Groningen, Netherlands.

Running title: Function and interactions in a minimal gut microbiome

Corresponding author: Willem M. de Vos; willem.devos@wur.nl

Supplementary Tables

Supplementary Table S1: Carbon sources used for growing pre-cultures of individual strains of DB-MM.

Sr. No.	Bacterial strain	Carbon sources (mM)
1	*Agathobacter rectalis* (DSM 17629)	Glucose (20 mM); lactose (20 mM); xylose (20 mM)
2	*Bacteroides ovatus 3_8_47FAA*	Glucose (20 mM); Galactose (20 mM); xylose (20 mM)
3	*Bacteroides sp 2_1_22*	Glucose (20 mM); Galactose (20 mM); xylose (20 mM)
4	*Coprococcus catus* ATCC® 27761™	Glucose (60 mM)
5	*Anaerobutyricum [Eubacterium] soehngenii* (DSM 17630)	Glucose (60 mM)
6	*Eubacterium siraeum* (DSM 15702)	Glucose (30 mM); (maltose 20 mM)
7	*Faecalibacterium prausnitzii* (A2-165)	Glucose (30 mM); fructose (30 mM)
8	Lachnospiraceae bacterium 7_1_58FAA (closely related to *F. plautii*)	Glucose (20 mM); fructose (20 mM)
9	*Roseburia intestinalis* (DSM 14610)	Glucose (30 mM); maltose (20 mM)
10	*Subdoligranulum variabile* (DSM 15176)	Glucose (60 mM)

Supplementary Table S2: Occupancy and metabolic features of the selected bacterial strains. *Weak growth reported previously. [β]barrnap predicted single short nucleotide sequence of 16S rRNA gene. [§]Information based on representative strains for each of the species. [#]The 16S rRNA gene copy numbers were calculated using the genome sequences.

Sr. No.	Bacterial strain	Strain used (16S rRNA gene copy number[#])	Occupancy (%)	Carbon source breakdown/utilization§, (reference)	NCBI Genome Accession
1	*Eubacterium rectale*	*Agathobacter [Eubacterium] rectalis* DSM 17629 (5)	97.4	Starch, cellobiose, inulin Butyrate and lactate production [1,2]	CP001107.1
2	*Bacteroides ovatus*	*Bacteroides ovatus* 3_8_47FAA (2)	92.4	Starch, xylan, inulin Acetate and propionate production [3]	GL945018.1
3	*Bacteroides xylanisolvens*	*Bacteroides* sp 2_1_22 (1)	76.1	Xylan, cellobiose [4]	GCF_000162155.1
4	*Coprococcus catus*	*Coprococcus catus* ATCC® 27761™ (1)	83.5	Lactate [5], fructose, glucose [6]	GCF_019734885.1
5	*Eubacterium hallii*	*Anaerobutyricum [Eubacterium] soehngenii* DSM 17630 (8)	92.3	Glucose, lactate plus acetate [7]	LT907978.1
6	*Eubacterium siraeum*	*Eubacterium siraeum* DSM 15702 (1)[β]	70.5	Starch*, xylan[8], fructose, cellobiose [9]	KB907512 ARNC01000000
7	*Faecalibacterium prausnitzii*	*Faecalibacterium prausnitzii* A2-165 (2)	99.2	Starch, inulin, pectin[10] [11]	GG697168.2
8	Lachnospiraceae bacterium 7_1_58FAA	Lachnospiraceae bacterium 7_1_58FAA (1)	90.2	Glucose, fructose	GCF_000242155.1
9	*Roseburia intestinalis*	*Roseburia intestinalis* DSM 14610 (3)	76.4	Starch, cellobiose*, fructose, xylan [12]	GCF_000156535.1
10	*Subdoligranulum unclassified*	*Subdoligranulum variabile* DSM 15176 (4)	99.8	Cellobiose, glucose [13]	GCF_000157955.1

Supplementary Table S3: Information regarding RNA-sequencing output. The total reads mapped to Db-MM refers to reads mapped after trimming, merging of paired-end reads and removal of rRNA reads.

Sample	Raw Reads	Clean Reads	Q20(%)	Q30(%)	GC Content (%)	Total reads mapped to Db-MM
B1T24	21796587	21400642	97.53	93.1	48.07	6841829
B2T24	23175032	22790250	97.56	93.06	47.14	8976800
B3T24	23539598	23165216	97.66	93.32	48.06	11104424
B1T48	20681656	20249822	97.59	93.24	51	10431307
B2T48	20401433	20090092	97.45	92.86	49.59	10489383
B3T48	22308228	21900920	97.55	93.12	49.77	12495581

Supplementary Table S4: Average nucleotide identity values comparing unclassified Db-MM strains with respective species type strains. We used the EzTaxon webserver's Average Nucleotide Identity (ANI) calculator tool [14,15].

Comparison	NCBI Genome Accession Reference vs Strain Genome	OrthoANIu value (%)
Flavonifractor.plautii - Lachnospiraceae bacterium 7_1_58FAA	NZ_CP048436.1 - GCF_000242155.1	98.15
Bacteroides.ovatus - Bacteroides.ovatus 3_8_47FAA	NZ_CP012938.1 - GL945018.1	96.75
B.xylanisolvens - Bacteroides sp 2_1_22	ASM1566878v1 - GCF_000162155.1	97.46

Supplementary Table S5: Enzymatic quantification of D and L- Lactate.

Sample	D-Lactate (mM)	L-Lactate (mM)
B1T24	0.69 ± 0.15	4.35 ± 0.24
B2T24	1.10 ± 0.03	5.10 ± 0.05
B3T24	3.41 ± 0.27	5.34 ± 0.28

Supplementary Table S6: Raw counts of Db-MM strains in metatranscriptomes of triplicate sample from 24 hr and 48 hr time points.

	24 hr			48 hr		
**Db-MM species**	**B1T24**	**B2T24**	**B3T24**	**B1T48**	**B2T48**	**B3T48**
Agathobacter rectalis	1189883	1533353	1994131	680440	1288745	1758023
Anaerobutyricum soehngenii	253483	385919	100388	153621	188132	462404
Bacteroides ovatus	396928	612919	1564701	664898	778758	1144162
Bacteroides xylanisolvens	415784	718469	1352381	627481	1064527	1337283
Coprococcus catus	268453	256730	97931	255466	210763	806083
Eubacterium siraeum	1705679	2685626	562719	1248773	1228419	738649
Faecalibacterium prausnitzii	1472613	1517503	3945749	4804605	3807111	3908967
Flavonifractor plautii	342532	359223	404157	1154960	994472	948667
Roseburia intestinalis	654577	755884	684482	259317	350652	349584
Subdoligranulum variabile	141897	151174	397785	581746	577804	1041759

Supplementary Table S7: Differential expression analysis of total transcript abundance mapped to individual bacteria strains in metatranscriptomics. The transcripts per million counts for each strain in the metatranscriptomic data were compared between 24 h and 48 h based on Wald test using DESeq2 package [16]. Significant values are highlighted as grey coloured cells.

**Bacteria**	**baseMean**	**FoldChange (log2)**	**lfcSE**	**Wald test p-value**	**Benjamini-Hochberg (BH) padj**
Faecalibacterium prausnitzii	3107078.28	0.72	0.42	0.0867890	0.1735780
Eubacterium siraeum	1478596.15	-0.92	0.67	0.1696240	0.2827070
Agathobacter rectalis	1368737.08	-0.66	0.31	0.0307960	0.0769910
Bacteroides xylanisolvens	855146.84	0.06	0.32	0.8395630	0.8395630
Subdoligranulum variabile	799716.82	-0.18	0.34	0.6038490	0.6709430
Bacteroides ovatus	680889.19	1.24	0.31	0.0000824	0.0002750
Flavonifractor plautii	530992.53	-1.43	0.27	0.0000002	0.0000017
Roseburia intestinalis	432720.22	1.44	0.31	0.0000042	0.0000212
Coprococcus catus	293997.97	0.46	0.59	0.4354750	0.6221070
Anaerobutyricum soehngenii	253901.51	-0.39	0.68	0.5676990	0.6709430

* lfcSE: standard error of the log2FoldChange

Supplementary Table S8: Community level fold changes in KO abundance in metatranscriptomics between 24 h and 48 h. The KEGG KOs that were identified by gene set enrichment analysis in supplementary figure S2.

**Sr. No.**	**KO ID**	**Fold change**	**Gene Name**
1	K00024	-2.94096	mdh; malate dehydrogenase [EC:1.1.1.37]
2	K00033	-2.89569	PGD, gnd, gntZ; 6-phosphogluconate dehydrogenase [EC:1.1.1.44 1.1.1.343]
3	K00036	-2.17893	G6PD, zwf; glucose-6-phosphate 1-dehydrogenase [EC:1.1.1.49 1.1.1.363]
4	K00053	-1.66609	ilvC; ketol-acid reductoisomerase [EC:1.1.1.86]
5	K00128	1.523144	ALDH; aldehyde dehydrogenase (NAD+) [EC:1.2.1.3]
6	K00170	2.492078	porB; pyruvate ferredoxin oxidoreductase beta subunit [EC:1.2.7.1]
7	K00171	3.300088	porD; pyruvate ferredoxin oxidoreductase delta subunit [EC:1.2.7.1]
8	K00174	-1.50009	korA, oorA, oforA; 2-oxoglutarate/2-oxoacid ferredoxin oxidoreductase subunit alpha [EC:1.2.7.3 1.2.7.11]
9	K00175	-1.57553	korB, oorB, oforB; 2-oxoglutarate/2-oxoacid ferredoxin oxidoreductase subunit beta [EC:1.2.7.3 1.2.7.11]
10	K00177	-1.63584	korC, oorC; 2-oxoglutarate ferredoxin oxidoreductase subunit gamma [EC:1.2.7.3]
11	K00198	1.54816	cooS, acsA; anaerobic carbon-monoxide dehydrogenase catalytic subunit [EC:1.2.7.4]
12	K00226	-2.12646	pyrD; dihydroorotate dehydrogenase (fumarate) [EC:1.3.98.1]
13	K00239	-2.08143	sdhA, frdA; succinate dehydrogenase / fumarate reductase, flavoprotein subunit [EC:1.3.5.1 1.3.5.4]
14	K00240	-1.99028	sdhB, frdB; succinate dehydrogenase / fumarate reductase, iron-sulfur subunit [EC:1.3.5.1 1.3.5.4]
15	K00241	-2.79632	sdhC, frdC; succinate dehydrogenase / fumarate reductase, cytochrome b subunit
16	K00425	-2.8396	cydA; cytochrome bd ubiquinol oxidase subunit I [EC:7.1.1.7]
17	K00426	-2.30804	cydB; cytochrome bd ubiquinol oxidase subunit II [EC:7.1.1.7]
18	K00605	-2.01977	gcvT, AMT; aminomethyltransferase [EC:2.1.2.10]
19	K00627	1.905094	DLAT, aceF, pdhC; pyruvate dehydrogenase E2 component (dihydrolipoamide acetyltransferase) [EC:2.3.1.12]
20	K00632	1.97916	fadA, fadI; acetyl-CoA acyltransferase [EC:2.3.1.16]
21	K00895	-1.73544	pfp, PFP; diphosphate-dependent phosphofructokinase [EC:2.7.1.90]
22	K00940	-2.85147	ndk, NME; nucleoside-diphosphate kinase [EC:2.7.4.6]

23	K00954	-1.71944	E2.7.7.3A, coaD, kdtB; pantetheine-phosphate adenylyltransferase [EC:2.7.7.3]
24	K01007	1.694605	pps, ppsA; pyruvate, water dikinase [EC:2.7.9.2]
25	K01029	2.235891	E2.8.3.5B, scoB; 3-oxoacid CoA-transferase subunit B [EC:2.8.3.5]
26	K01057	-1.89191	PGLS, pgl, devB; 6-phosphogluconolactonase [EC:3.1.1.31]
27	K01176	-2.40483	AMY, amyA, malS; alpha-amylase [EC:3.2.1.1]
28	K01193	3.750748	INV, sacA; beta-fructofuranosidase [EC:3.2.1.26]
29	K01200	-1.80797	pulA; pullulanase [EC:3.2.1.41]
30	K01596	-2.50671	E4.1.1.32, pckA, PCK; phosphoenolpyruvate carboxykinase (GTP) [EC:4.1.1.32]
31	K01676	-1.75216	E4.2.1.2A, fumA, fumB; fumarate hydratase, class I [EC:4.2.1.2]
32	K01838	-2.47437	pgmB; beta-phosphoglucomutase [EC:5.4.2.6]
33	K01847	-1.66061	MUT; methylmalonyl-CoA mutase [EC:5.4.99.2]
34	K01848	2.859385	E5.4.99.2A, mcmA1; methylmalonyl-CoA mutase, N-terminal domain [EC:5.4.99.2]
35	K01849	2.476116	E5.4.99.2B, mcmA2; methylmalonyl-CoA mutase, C-terminal domain [EC:5.4.99.2]
36	K01902	-1.54862	sucD; succinyl-CoA synthetase alpha subunit [EC:6.2.1.5]
37	K01960	-2.31476	pycB; pyruvate carboxylase subunit B [EC:6.4.1.1]
38	K02106	1.96424	atoE; short-chain fatty acids transporter
39	K02387	-1.55908	flgB; flagellar basal-body rod protein FlgB
40	K02388	-1.70273	flgC; flagellar basal-body rod protein FlgC
41	K02390	-1.50895	flgE; flagellar hook protein FlgE
42	K02406	-2.69061	fliC; flagellin
43	K02407	-2.07865	fliD; flagellar hook-associated protein 2
44	K02408	-1.56432	fliE; flagellar hook-basal body complex protein FliE
45	K02414	-2.38734	fliK; flagellar hook-length control protein FliK
46	K02422	-2.16507	fliS; flagellar protein FliS
47	K02437	-1.98741	gcvH, GCSH; glycine cleavage system H protein
48	K02650	-2.79189	pilA; type IV pilus assembly protein PilA
49	K03389	1.795722	hdrB2; heterodisulfide reductase subunit B2 [EC:1.8.7.3 1.8.98.4 1.8.98.5 1.8.98.6]
50	K03407	-1.67972	cheA; two-component system, chemotaxis family, sensor kinase CheA [EC:2.7.13.3]
51	K03408	-1.69399	cheW; purine-binding chemotaxis protein CheW
52	K03412	-1.58349	cheB; two-component system, chemotaxis family, protein-glutamate methylesterase/glutaminase [EC:3.1.1.61 3.5.1.44]
53	K03415	-1.72794	cheV; two-component system, chemotaxis family, chemotaxis protein CheV
54	K03781	-2.32585	katE, CAT, catB, srpA; catalase [EC:1.11.1.6]
55	K05341	4.106092	E2.4.1.4; amylosucrase [EC:2.4.1.4]
56	K06016	1.972509	pydC; beta-ureidopropionase / N-carbamoyl-L-amino-acid hydrolase [EC:3.5.1.6 3.5.1.87]
57	K06896	1.500823	mapP; maltose 6'-phosphate phosphatase [EC:3.1.3.90]
58	K07250	1.990253	gabT; 4-aminobutyrate aminotransferase / (S)-3-amino-2-methylpropionate transaminase / 5-aminovalerate transaminase [EC:2.6.1.19 2.6.1.22 2.6.1.48]
59	K07405	-1.92605	E3.2.1.1A; alpha-amylase [EC:3.2.1.1]
60	K07652	1.897802	vicK; two-component system, OmpR family, sensor histidine kinase VicK [EC:2.7.13.3]
61	K07668	1.571842	vicR; two-component system, OmpR family, response regulator VicR
62	K07777	-2.06544	degS; two-component system, NarL family, sensor histidine kinase DegS [EC:2.7.13.3]
63	K08722	1.750147	yfbR; 5'-deoxynucleotidase [EC:3.1.3.89]
64	K10540	-1.79591	mglB; methyl-galactoside transport system substrate-binding protein
65	K10914	4.889803	crp; CRP/FNR family transcriptional regulator, cyclic AMP receptor protein
66	K11261	1.754817	fwdE, fmdE; formylmethanofuran dehydrogenase subunit E [EC:1.2.7.12]
67	K11645	-1.86848	fbaB; fructose-bisphosphate aldolase, class I [EC:4.1.2.13]

68	K17722	5.06483	preT; dihydropyrimidine dehydrogenase (NAD+) subunit PreT [EC:1.3.1.1]
69	K17723	1.884507	preA; dihydropyrimidine dehydrogenase (NAD+) subunit PreA [EC:1.3.1.1]
70	K18118	2.62769	aarC, cat1; succinyl-CoA:acetate CoA-transferase [EC:2.8.3.18]
71	K18344	1.579435	vanRB, vanR, vanRD; two-component system, OmpR family, response regulator VanR
72	K18348	2.592029	vanT; serine/alanine racemase [EC:5.1.1.18 5.1.1.1]
73	K18856	2.704648	vanC, vanE, vanG; D-alanine---D-serine ligase [EC:6.3.2.35]
74	K18866	2.642704	vanXY; zinc D-Ala-D-Ala dipeptidase/carboxypeptidase [EC:3.4.13.22 3.4.17.14]
75	K20489	2.340271	nisI; lantibiotic immunity protein
76	K22373	-2.31388	larA; lactate racemase [EC:5.1.2.1]
77	K23259	-2.14437	adh; isopropanol dehydrogenase (NADP+) [EC:1.1.1.80]

Supplementary Figures

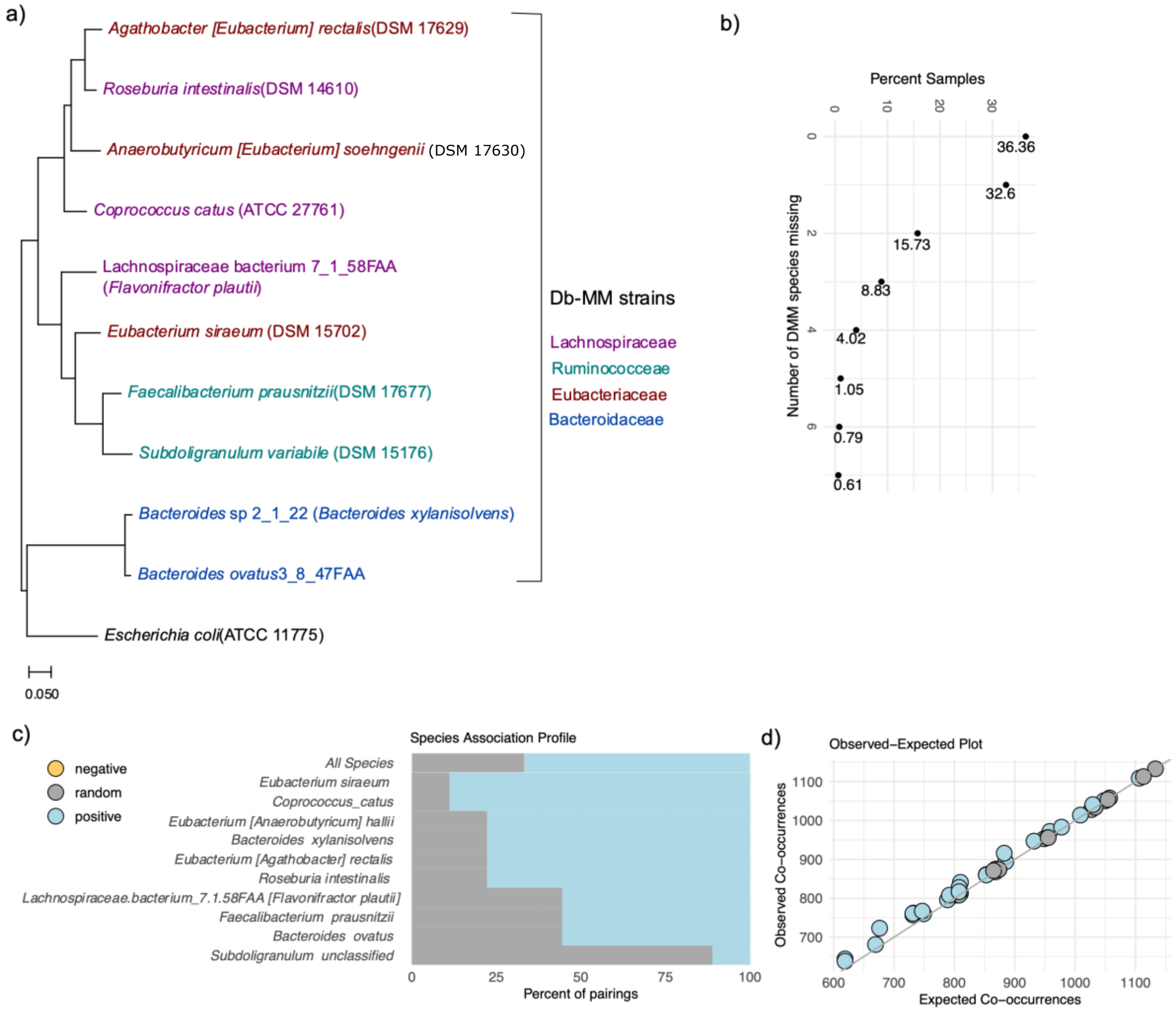

Supplementary Figure S1: Phylogeny and co-occurrences of Db-MM strains in human gut metagenomes. a) 16S rRNA gene based phylogenetic relationship inferred using the Maximum Likelihood method and Kimura 2-parameter model. The analysis was done using MEGA11[17,18]. *E. coli* is used as outgroup for building the tree and is not part of the Db-MM. The taxonomic affiliation for DB-MM strains is based on curatedmetagenomicDB data that we used in our study. b) Percent of samples out of 1144 human gut metagenomes in which Db-MM species are observed. We observed the 36 % of samples consisted all ten Db-MM species. c) Co-occurrence profiles for Db-MM species in 1144 human gut metagenomes determined using the probabilistic co-occurrence model. d) Expected co-occurrence profiles for Db-MM species in 1144 human gut metagenomes compared with the null-model.

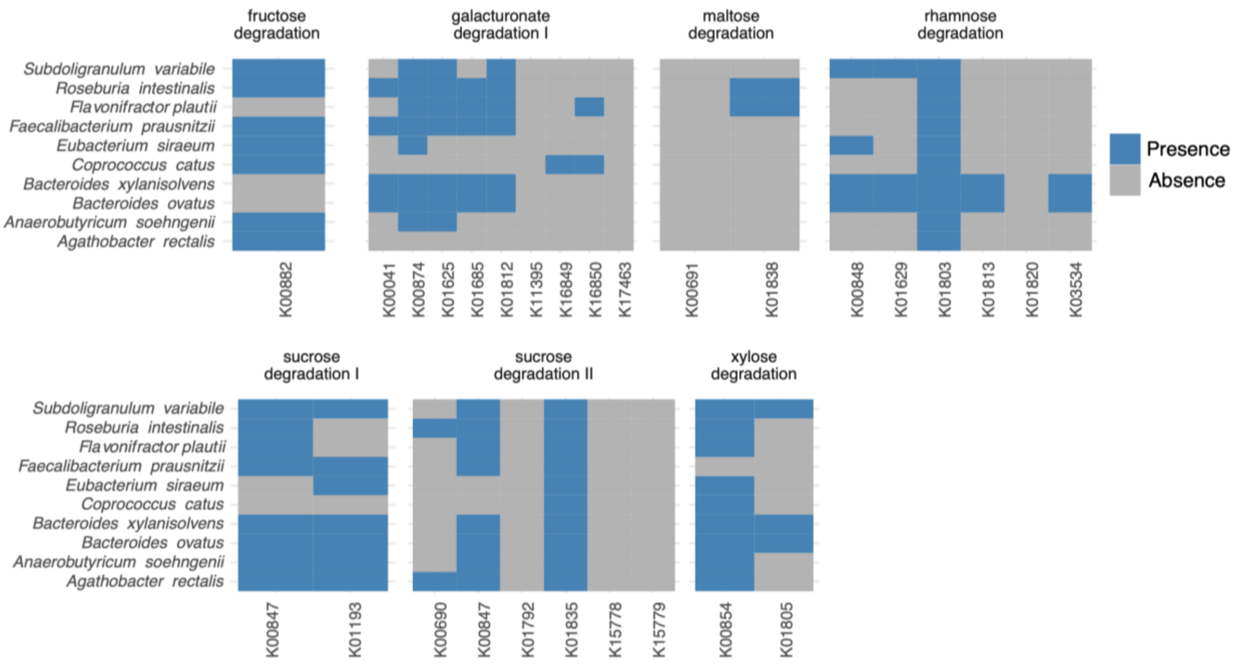

Supplementary Figure S2: Genomic potential of Db-MM species for degradation of monosaccharides, sucrose and galacturonate. Presence of specific KEGG orthologs defined by gut metabolic modules was investigated in genomes of strains used in Db-MM.

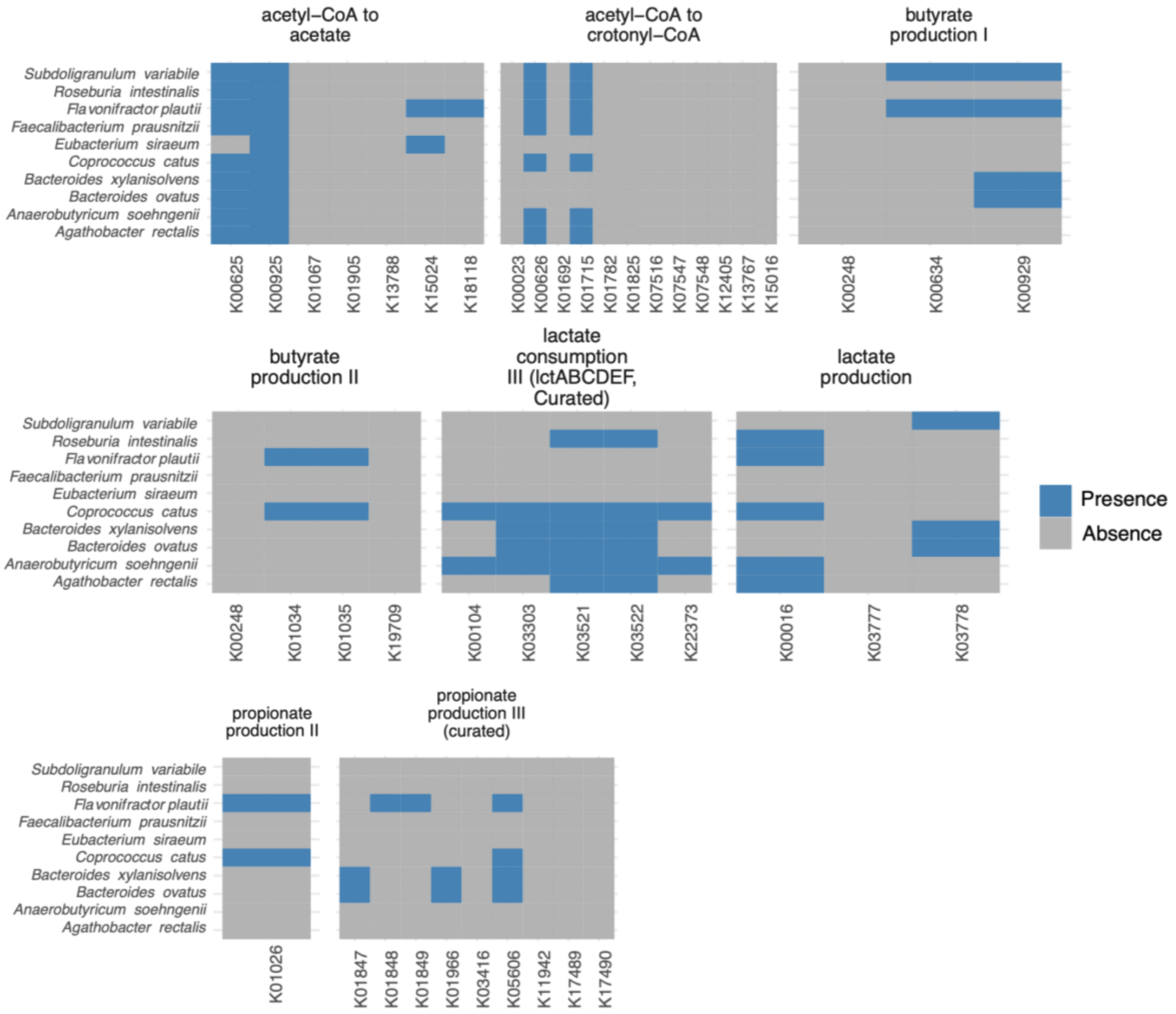

Supplementary Figure S3: Genomic potential of Db-MM species for fermentative metabolism. Presence of specific KEGG orthologs defined by gut metabolic modules was investigated in genomes of strains used in Db-MM.

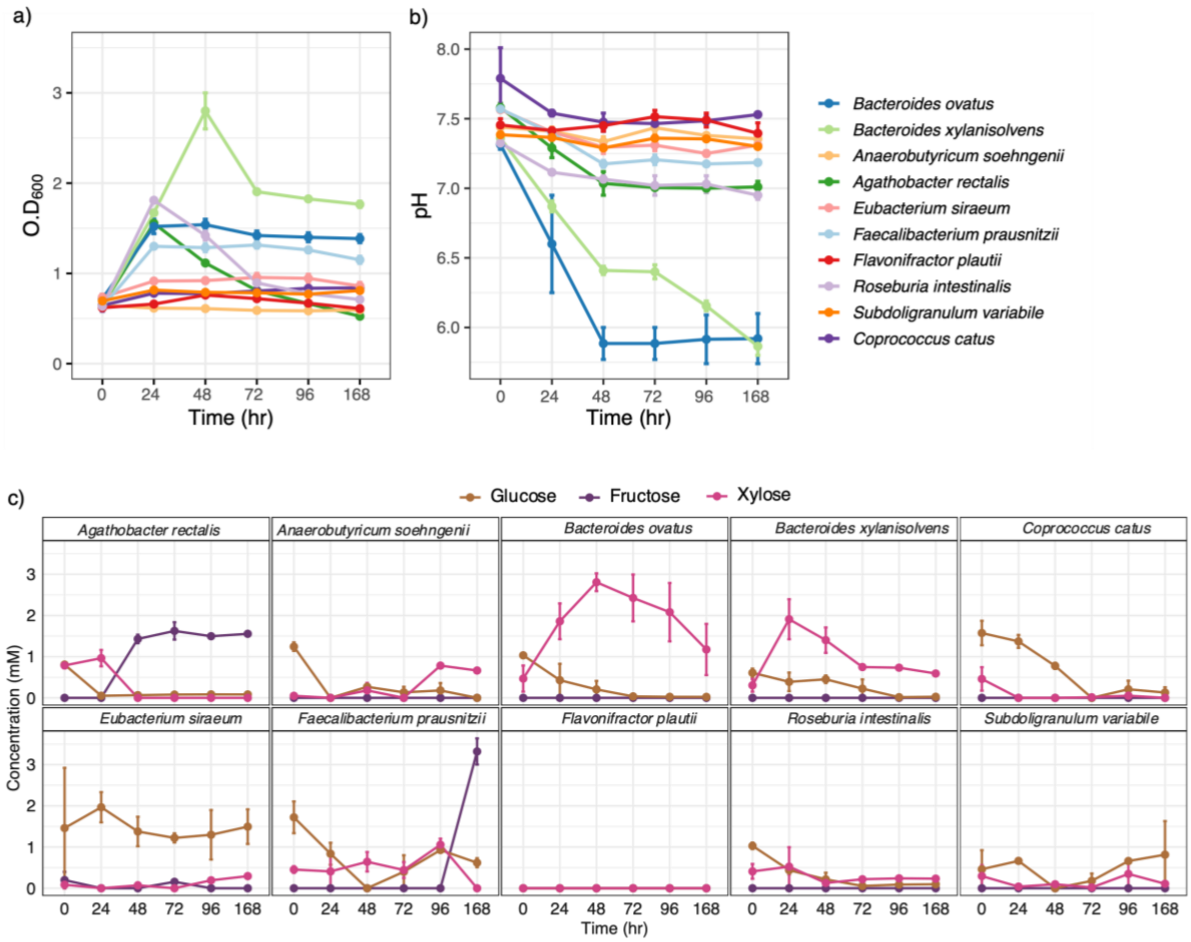

Supplementary Figure S4: Mono-culture observations for Db-MM species. a) Growth of Db-MM species in mono-culture measures as $O.D_{600}$ in presence of mixture of pectin, xylan, starch, inulin and cellobiose. B) Changes in pH for each Db-MM species when grown in mono-culture. c) Consumptions of mono-saccharides. Here, we detected minor amounts as these are affected by how the poly- and/or oligo- saccharides are degraded and consumed by each of the species.

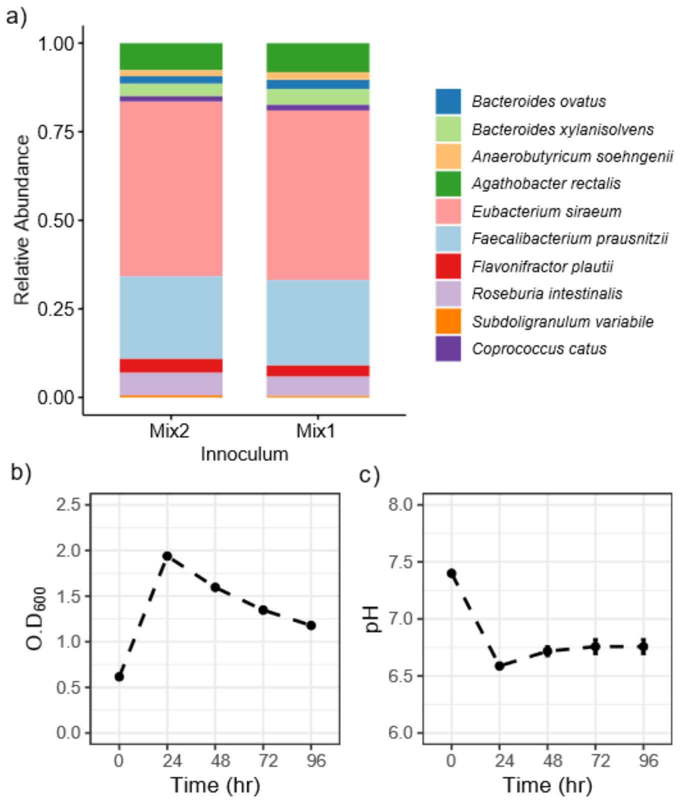

Supplementary Figure S5: Inoculum and growth of Db-MM. a) Composition of the inoculum used for assembling the Db-MM. Composition was measured using 16S rRNA gene sequencing and copy number corrected abundances are visualized as barplot. b) Optical density ($O.D_{600}$) of Db-MM. c) pH changes during growth of Db-MM.

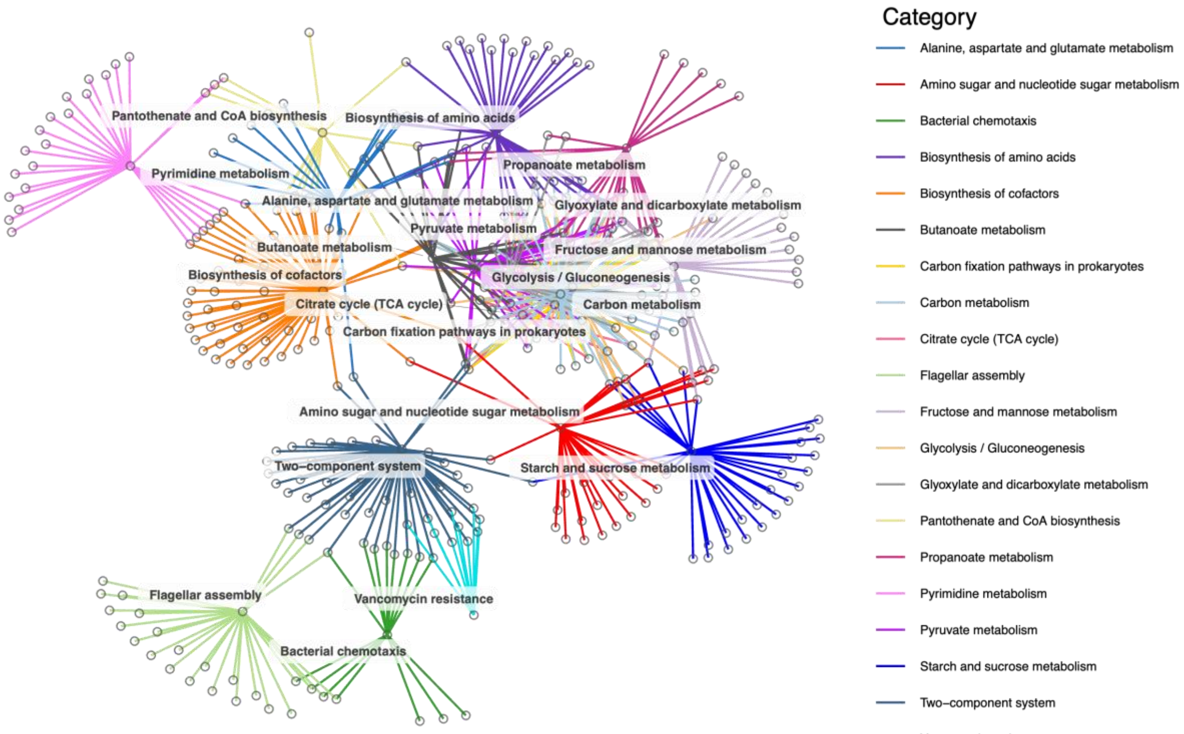

Supplementary Figure S6: Metabolic interaction network in Db-MM. Gene-Concept Network depicting functional modules with overlapping gene sets connected via edges. The fold change for KEGG KO was calculated using DESeq2 R package were used to identify enriched gene (KO) sets and pathway categories [15,16].

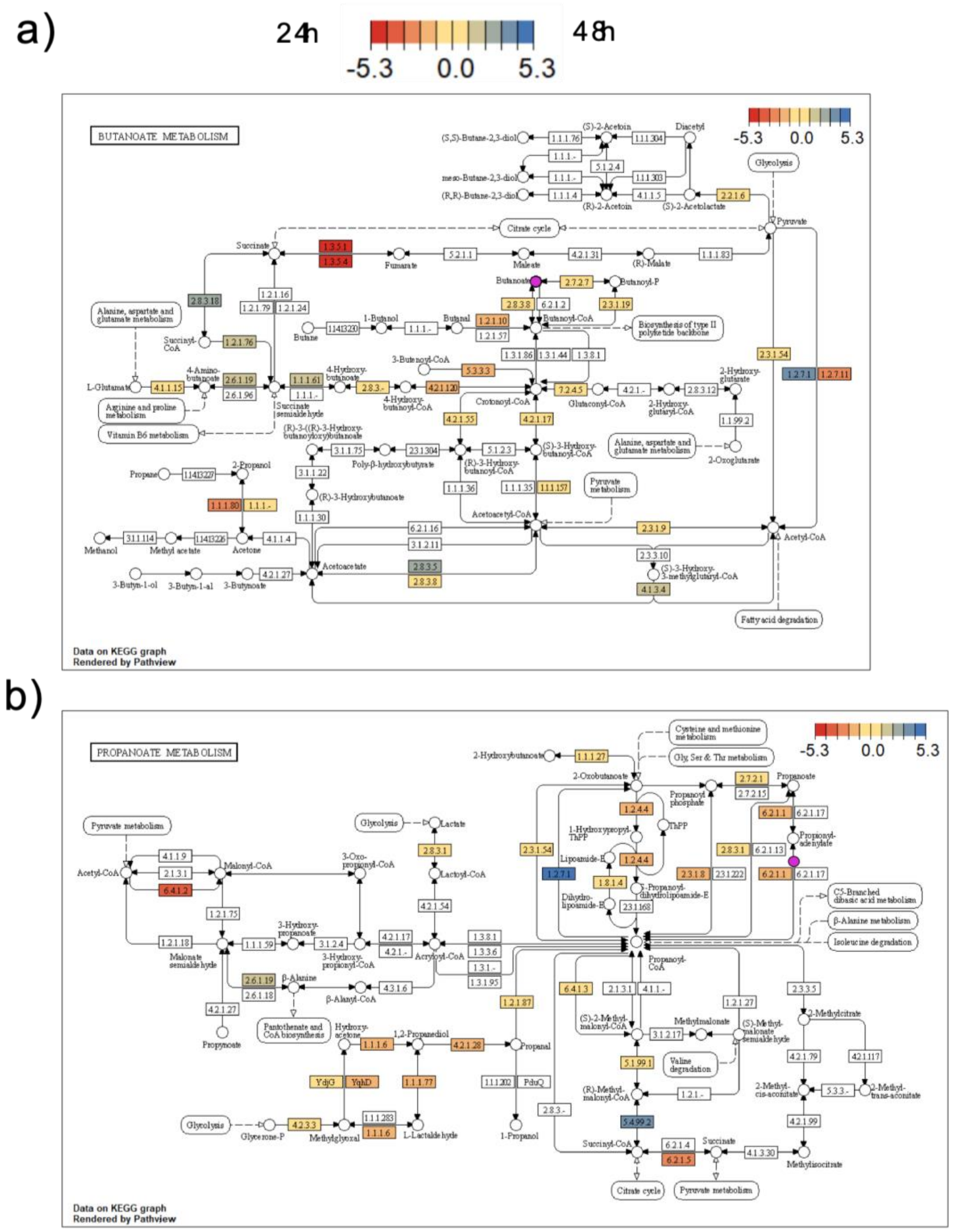

Supplementary Figure S7: Reconstructed metabolic pathways in the Db-MM. a) Butanoate (butyrate; purple filled circle) metabolism b) Propanoate (propionate; purple filled circle) metabolism. The reconstructed active gut metabolic modules in metatranscriptomics data were mapped on KEGG overviews of metabolic pathways using pathview. Red colour indicates higher expression at 24 h (n=3) and blue indicates higher expression at 48 h (n=3).

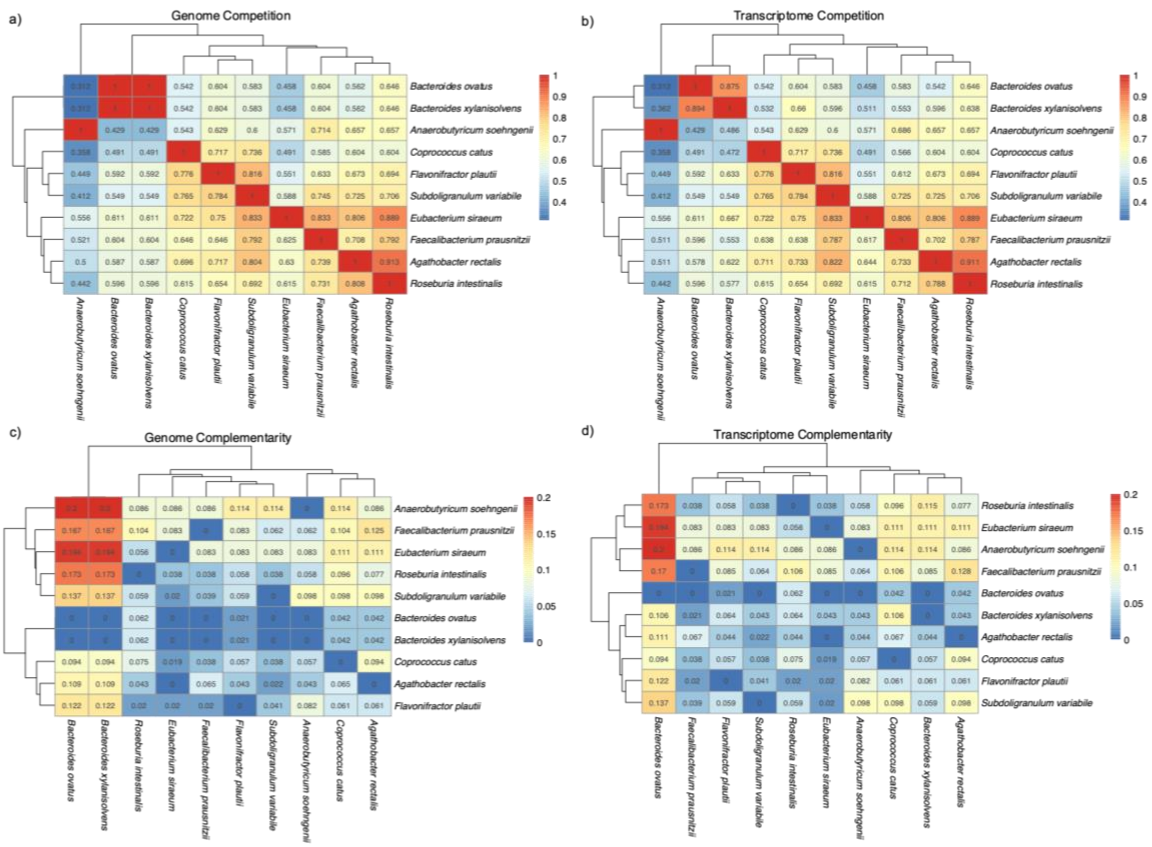

Supplementary Figure S8: Pair-wise interactions predicted using reverse ecology approach. a) and c) are based on KEGG KOs derived metabolic network based the genomes of each of the strains while b) and d) are based on KEGG KOs derived metabolic network based on strain-level gene expression in metatranscriptomic data.

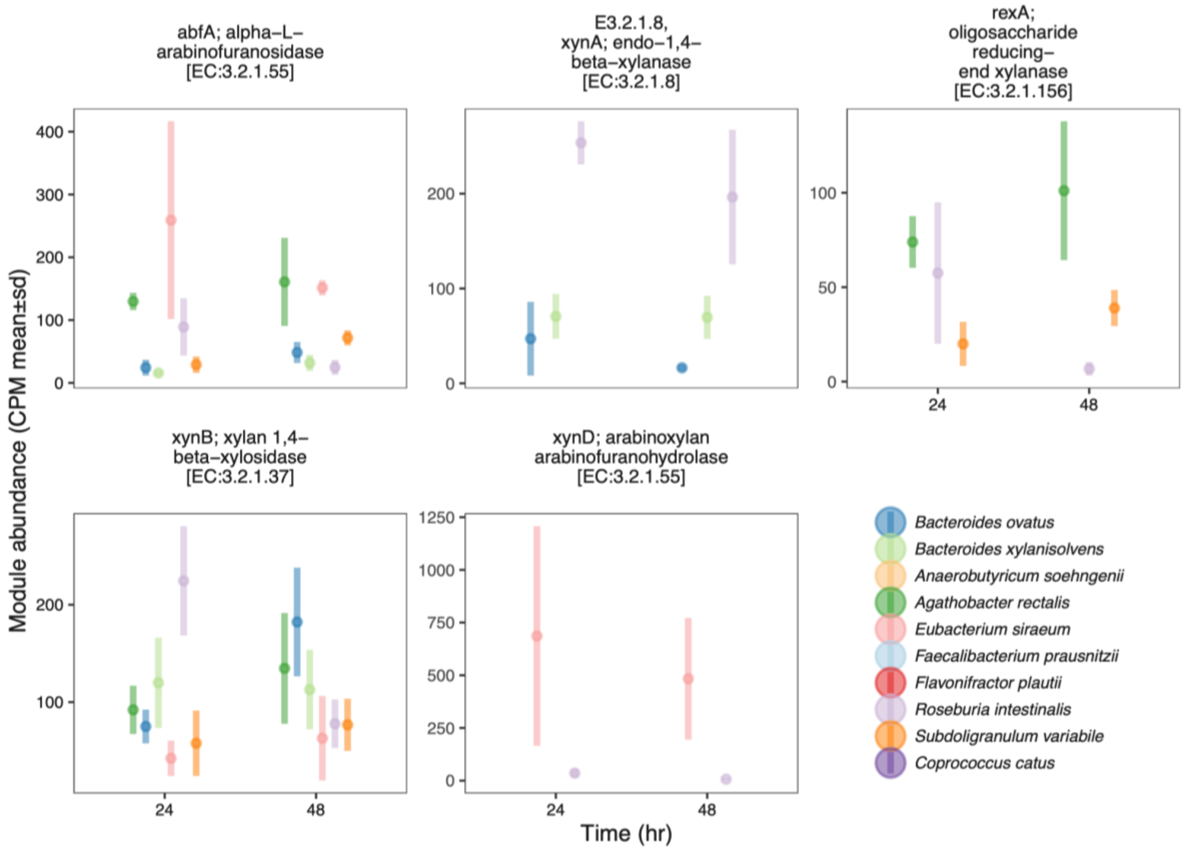

Supplementary Figure S9: Abundances of KEGG KOs part of arabinoxylan degradation module. The points represent the mean values and bars represent standard deviation values for each of the replicate.

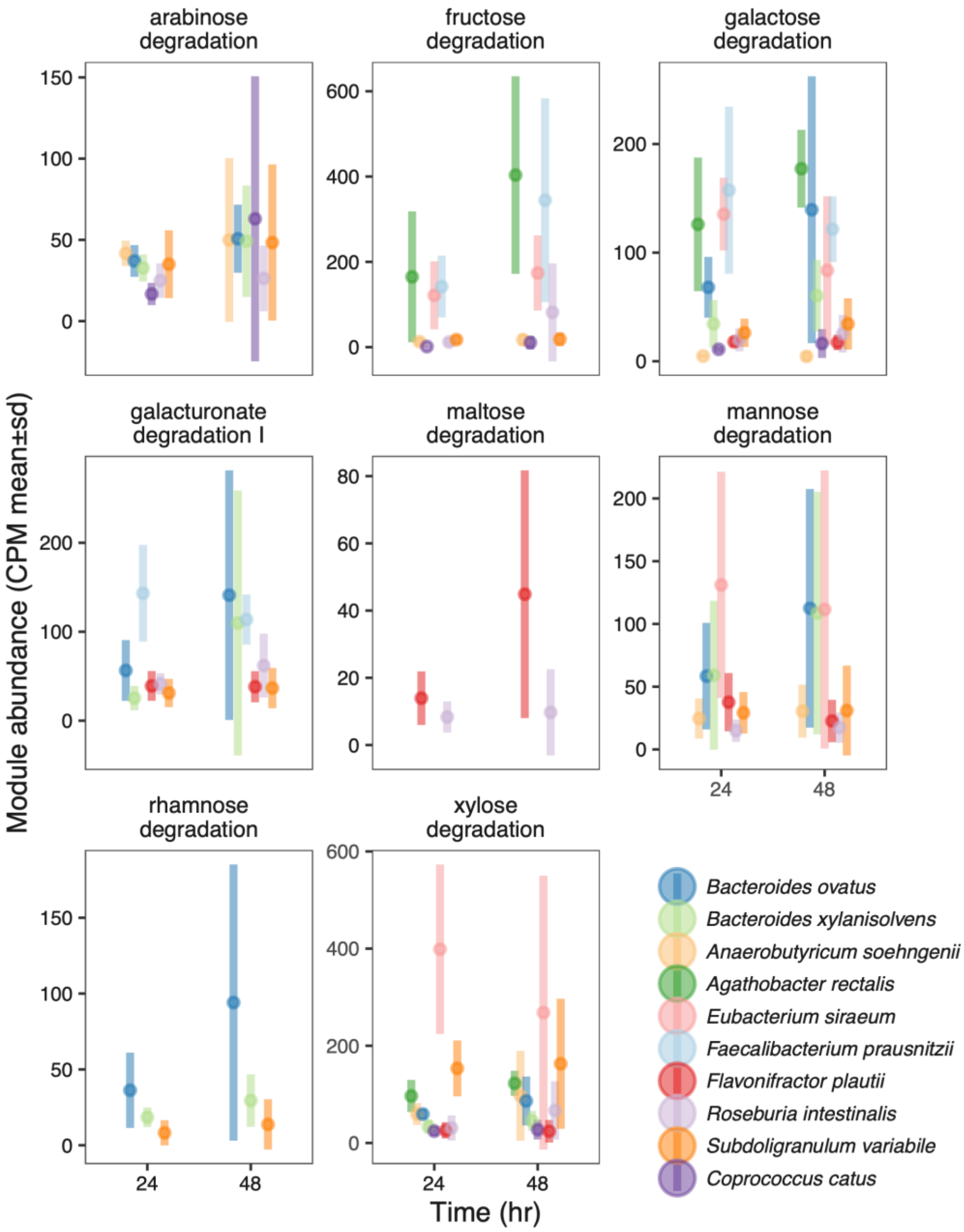

Supplementary Figure S10: Species specific abundances of metabolic modules for mono-, di-saccharides and galacturonate. The points represent the mean values and bars represent standard deviation values for each of the replicates.

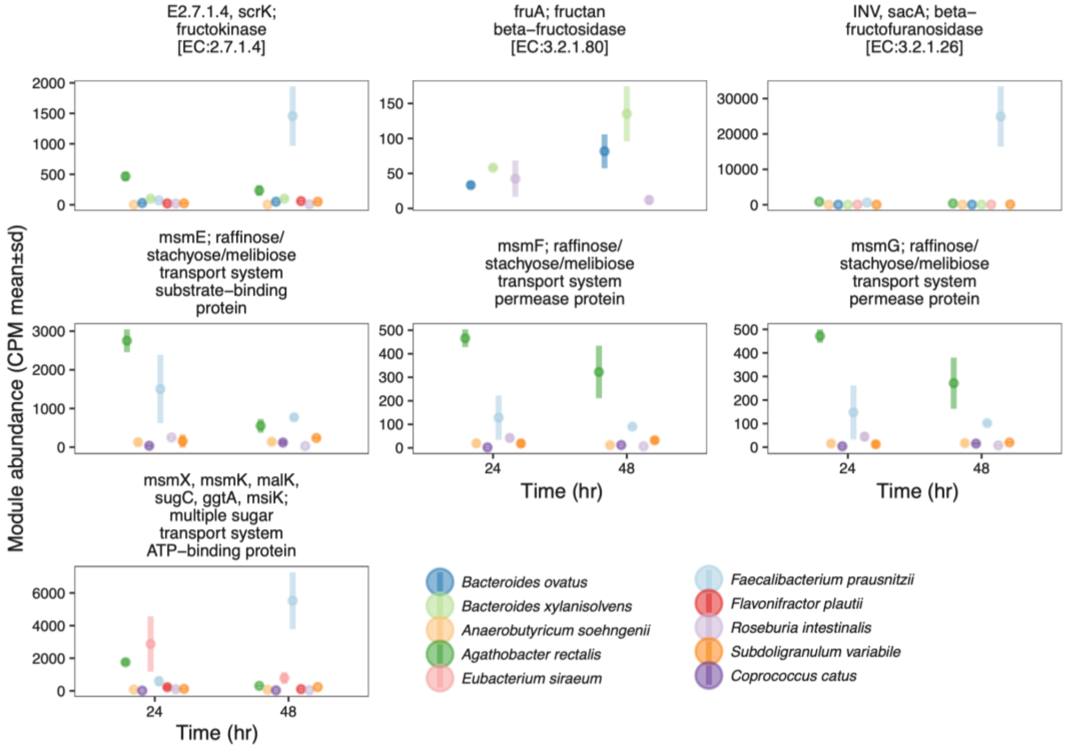

Supplementary Figure S11: Abundances of KEGG KOs part of fructan degradation module. The points represent the mean values and bars represent standard deviation values for each of the replicate.

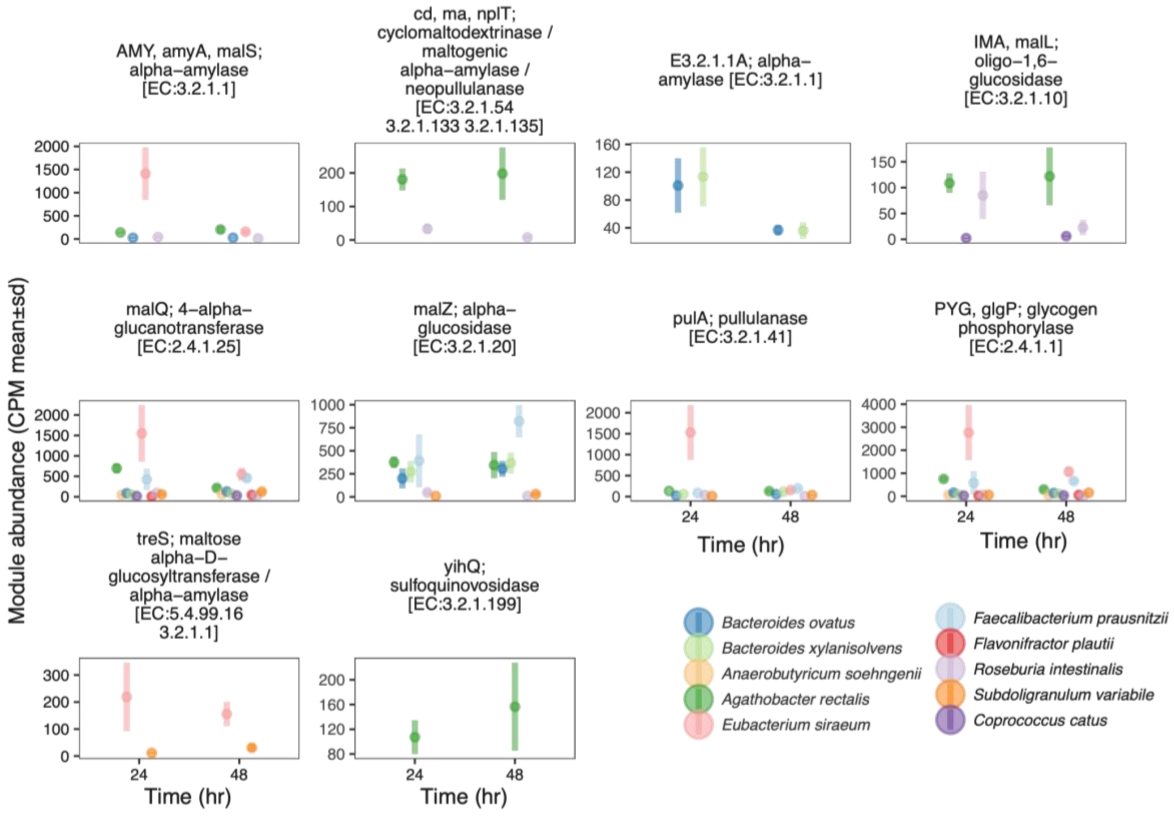

Supplementary Figure S12: Abundances of KEGG KOs part of starch degradation module. The points represent the mean values and bars represent standard deviation values for each of the replicate.

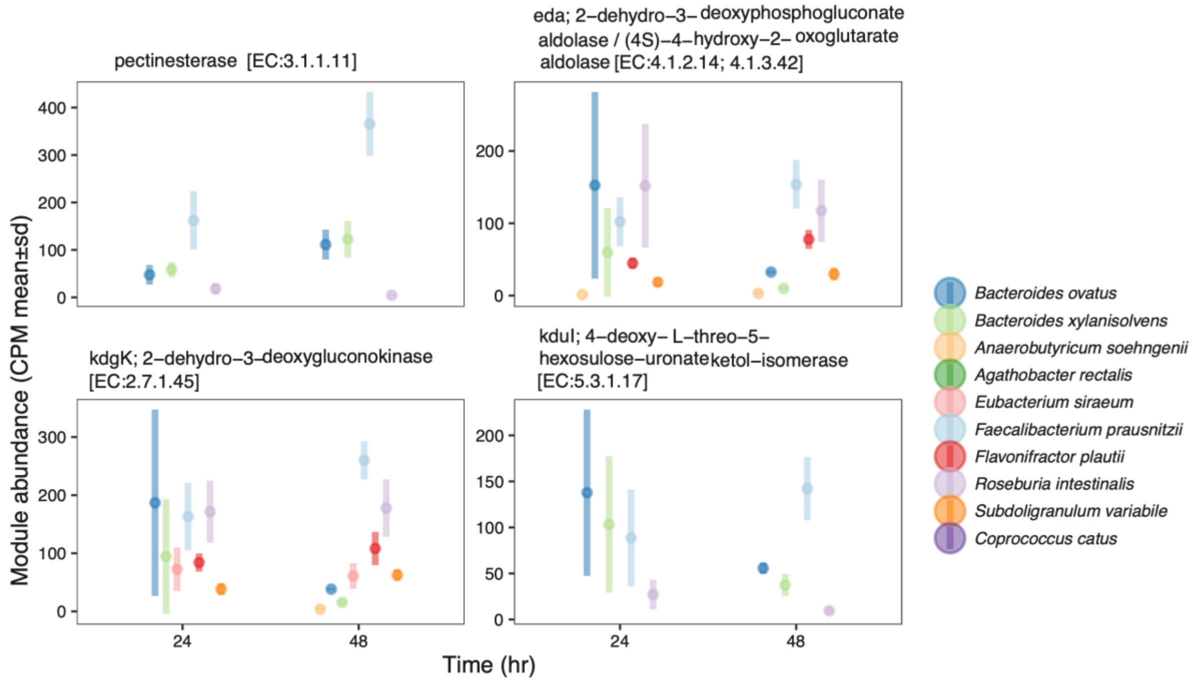

Supplementary Figure S13: Abundances of KEGG KOs part of pectin degradation module. The points represent the mean values and bars represent standard deviation values for each of the replicate.

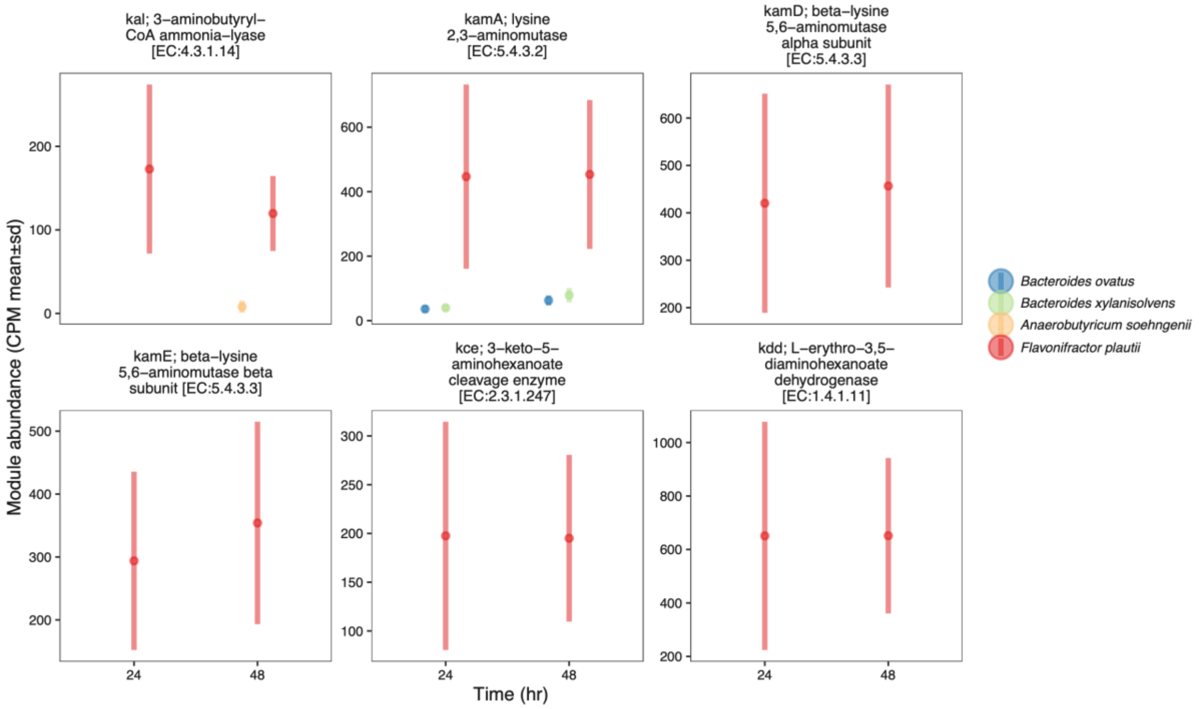

Supplementary Figure S14: Abundances of KEGG KOs in lysine degradation GMM. The points represent the mean values and bars represent standard deviation values for each of the replicate.

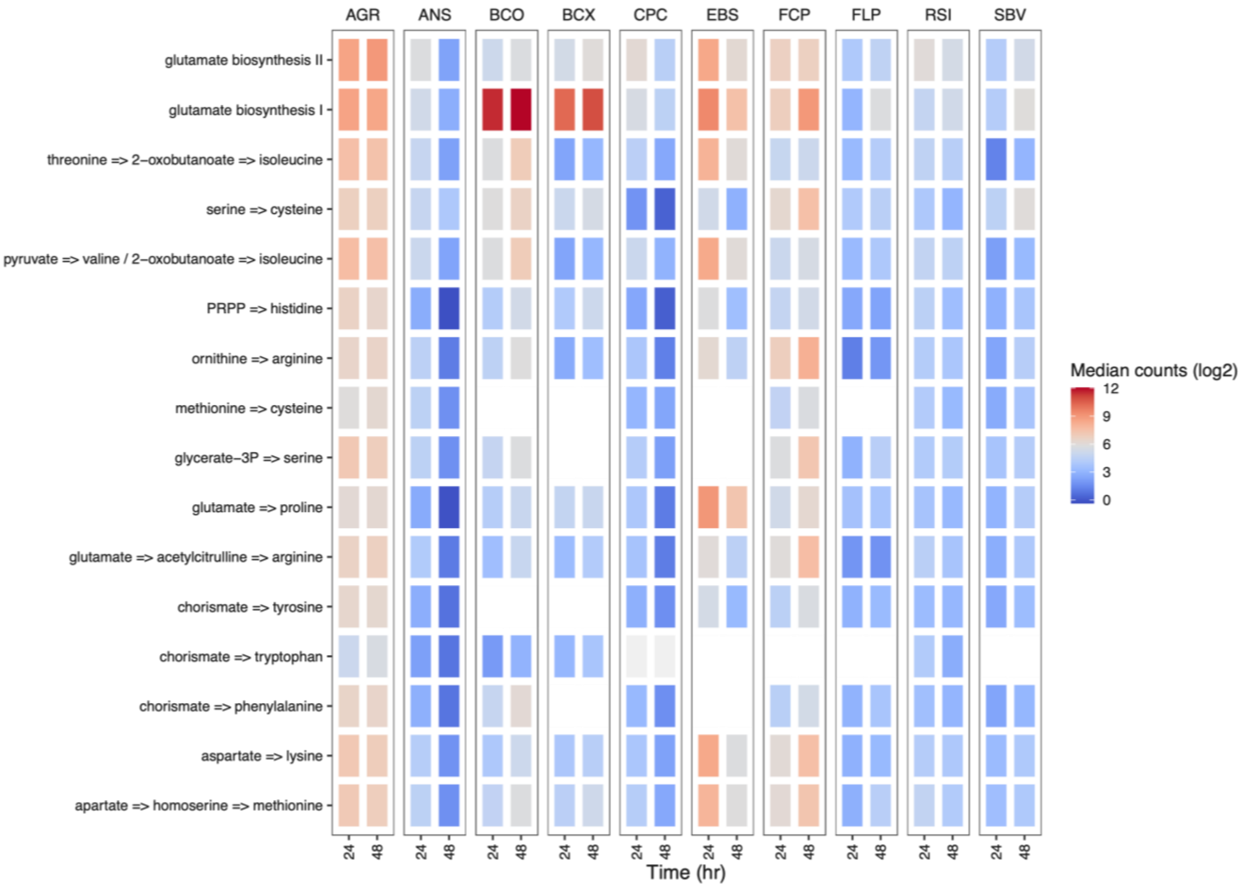

Supplementary Figure S15: Amino acid biosynthesis modules. The abundance values are counts per million log2 transformed for visualization. Abbreviations for taxon are: AGR, *A. rectalis*; ANS, *A. soehngenii*; BCO, *B. ovatus*; BCX, *B. xylanisolvens*; CPC, *C. catus*; EBS, *E. siraeum*; FCP, *F. prausnitzii*; FLP, *F. plautii*; RSI, *R. intestinalis*; SBV, *S. variabile*.

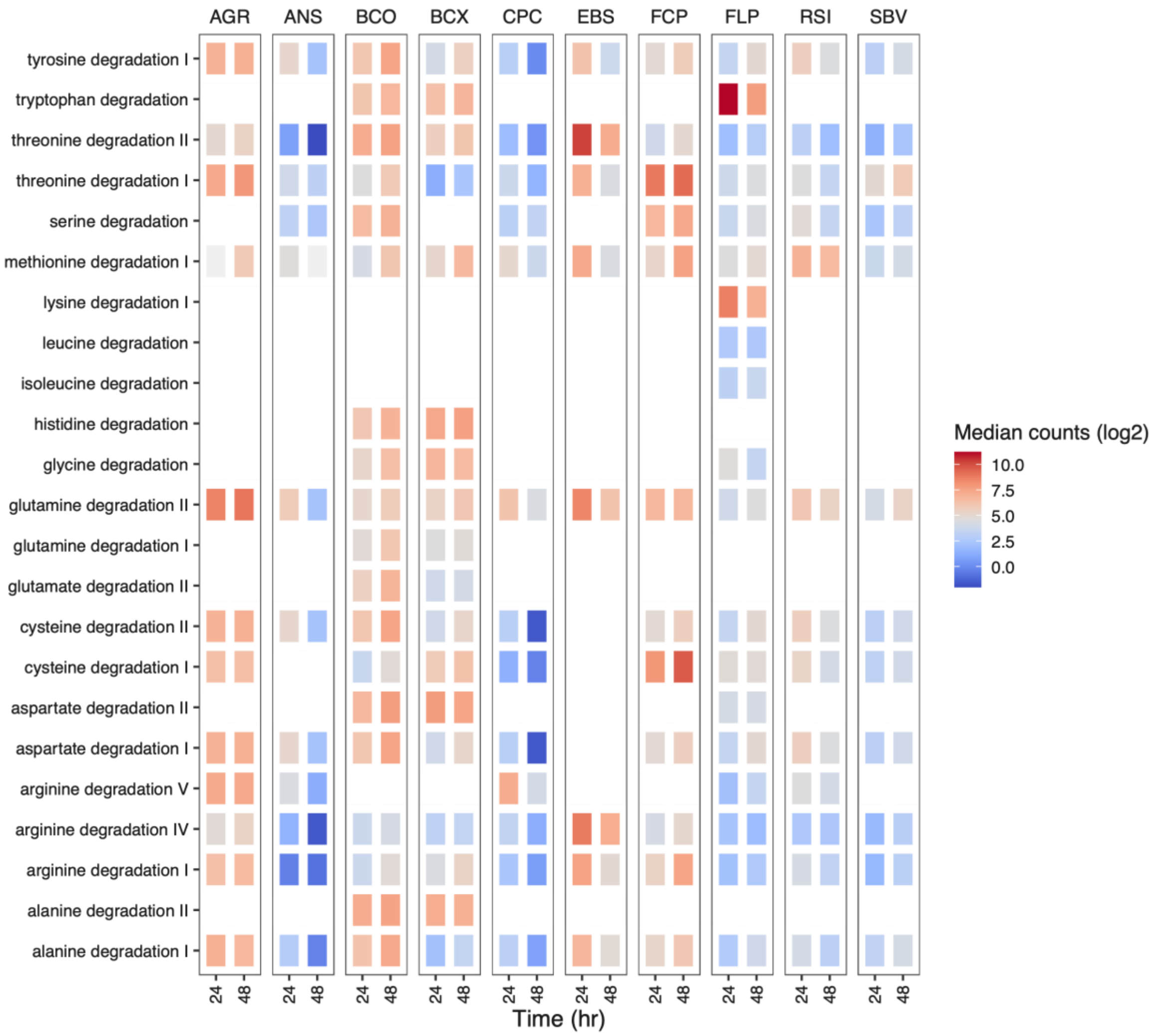

Supplementary Figure S16: Amino acid degradation modules. The abundance values are counts per million log2 transformed for visualization. Abbreviations for taxon are: AGR, *A. rectalis*; ANS, *A. soehngenii*; BCO, *B. ovatus*; BCX, *B. xylanisolvens*; CPC, *C. catus*; EBS, *E. siraeum*; FCP, *F. prausnitzii*; FLP, *F. plautii*; RSI, *R. intestinalis*; SBV, *S. variabile*.

Supplementary references

1 Cockburn, D. W. *et al.* Molecular details of a starch utilization pathway in the human gut symbiont *Eubacterium rectale*. *Molecular microbiology* **95**, 209-230 (2015).

2 Duncan, S. H. & Flint, H. J. Proposal of a neotype strain (A1-86) for *Eubacterium rectale*. Request for an opinion. *International journal of systematic and evolutionary microbiology* **58**, 1735-1736 (2008).

3 Sheridan, P. *et al.* Polysaccharide utilization loci and nutritional specialization in a dominant group of butyrate-producing human colonic Firmicutes. *Microbial Genomics* **2**, doi:doi:10.1099/mgen.0.000043 (2016).

4 Chassard, C., Delmas, E., Lawson, P. A. & Bernalier-Donadille, A. Bacteroides xylanisolvens sp. nov., a xylan-degrading bacterium isolated from human faeces. *International journal of systematic and evolutionary microbiology* **58**, 1008-1013 (2008).

5 Reichardt, N. *et al.* Phylogenetic distribution of three pathways for propionate production within the human gut microbiota. *The ISME journal* **8**, 1323 (2014).

6 Holdeman, L. V. & Moore, W. New genus, *Coprococcus*, twelve new species, and emended descriptions of four previously described species of bacteria from human feces. *International Journal of Systematic and Evolutionary Microbiology* **24**, 260-277 (1974).

7 Engels, C., Ruscheweyh, H.-J., Beerenwinkel, N., Lacroix, C. & Schwab, C. The common gut microbe Eubacterium hallii also contributes to intestinal propionate formation. *Frontiers in microbiology* **7**, 713 (2016).

8 Duncan, S. H. *et al.* Wheat bran promotes enrichment within the human colonic microbiota of butyrate-producing bacteria that release ferulic acid. *Environmental microbiology* **18**, 2214-2225 (2016).

9 MOORE, W. C., Johnson, J. & Holdeman, L. Emendation of Bacteroidaceae and Butyrivibrio and descriptions of Desulfomonas gen. nov. and ten new species in the genera Desulfomonas, Butyrivibrio, Eubacterium, Clostridium, and Ruminococcus. *International journal of systematic and evolutionary microbiology* **26**, 238-252 (1976).

10 Chung, W. S. F. *et al.* Prebiotic potential of pectin and pectic oligosaccharides to promote anti-inflammatory commensal bacteria in the human colon. *FEMS microbiology ecology* **93**, fix127 (2017).

11 Duncan, S. H., Hold, G. L., Harmsen, H. J., Stewart, C. S. & Flint, H. J. Growth requirements and fermentation products of Fusobacterium prausnitzii, and a proposal to reclassify it as Faecalibacterium prausnitzii gen. nov., comb. nov. *International journal of systematic and evolutionary microbiology* **52**, 2141-2146 (2002).

12 Duncan, S. H., Hold, G. L., Barcenilla, A., Stewart, C. S. & Flint, H. J. *Roseburia intestinalis* sp. nov., a novel saccharolytic, butyrate-producing bacterium from human faeces. *International journal of systematic and evolutionary microbiology* **52**, 1615-1620 (2002).

13 Holmstrøm, K., Collins, M. D., Møller, T., Falsen, E. & Lawson, P. A. Subdoligranulum variabile gen. nov., sp. nov. from human feces. *Anaerobe* **10**, 197-203 (2004).

14 Yoon, S.-H., Ha, S.-M., Lim, J., Kwon, S. & Chun, J. A large-scale evaluation of algorithms to calculate average nucleotide identity. *Antonie Van Leeuwenhoek* **110**, 1281-1286 (2017).

15 Chun, J. *et al.* EzTaxon: a web-based tool for the identification of prokaryotes based on 16S ribosomal RNA gene sequences. *International journal of systematic and evolutionary microbiology* **57**, 2259-2261 (2007).

16 Love, M. I., Huber, W. & Anders, S. Moderated estimation of fold change and dispersion for RNA-seq data with DESeq2. *Genome biology* **15**, 550 (2014).

17 Kimura, M. A simple method for estimating evolutionary rates of base substitutions through comparative studies of nucleotide sequences. *Journal of molecular evolution* **16**, 111-120 (1980).

18 Tamura, K., Stecher, G. & Kumar, S. MEGA11: molecular evolutionary genetics analysis version 11. *Molecular Biology and Evolution* **38**, 3022-3027 (2021).
